# Globalization and pollution: tele-connecting local primary PM_2.5_ emissions to global consumption

**DOI:** 10.1098/rspa.2016.0380

**Published:** 2016-11

**Authors:** Jing Meng, Junfeng Liu, Yuan Xu, Dabo Guan, Zhu Liu, Ye Huang, Shu Tao

**Affiliations:** 1 Laboratory for Earth Surface Processes, College of Urban and Environmental Sciences, Peking University, Beijing, People's Republic of China; 2 School of Environmental Sciences, University of East Anglia, Norwich, NR4 7JT, UK; 3 Department of Geography and Resource Management and Institute of Environment, Energy and Sustainability, The Chinese University of Hong Kong, Hong Kong, People's Republic of China; 4 School of International Development, University of East Anglia, Norwich NR4 7TJ, UK; 5 Resnick Sustainability Institute, California Institute of Technology, Pasadena, CA 91125, USA; 6 Laboratoire des Sciences du Climat et de l'Environnement, Commissariat à l'Energie Atomique-Centre National de la Recherche Scientifique-Université de Versailles Saint-Quentin-en-Yvelines, Centre d'Etudes Orme des Merisiers, 91191 Gif sur Yvette, France

**Keywords:** PM_2.5_ emissions, multi-region input–output analysis, globalization

## Abstract

Globalization pushes production and consumption to geographically diverse locations and generates a variety of sizeable opportunities and challenges. The distribution and associated effects of short-lived primary fine particulate matter (PM_2.5_), a representative of local pollution, are significantly affected by the consumption through global supply chain. Tele-connection is used here to represent the link between production and consumption activity at large distances. In this study, we develop a global consumption-based primary PM_2.5_ emission inventory to track primary PM_2.5_ emissions embodied in the supply chain and evaluate the extent to which local PM_2.5_ emissions are triggered by international trade. We further adopt consumption-based accounting and identify the global original source that produced the emissions. We find that anthropogenic PM_2.5_ emissions from industrial sectors accounted for 24 Tg globally in 2007; approximately 30% (7.2 Tg) of these emissions were embodied in export of products principally from Brazil, South Africa, India and China (3.8 Tg) to developed countries. Large differences (up to 10 times) in the embodied emissions intensity between net importers and exporters greatly increased total global PM_2.5_ emissions. Tele-connecting production and consumption activity provides valuable insights with respect to mitigating long-range transboundary air pollution and prompts concerted efforts aiming at more environmentally conscious globalization.

## 1. Introduction

In a highly globalized world, goods and services are frequently not consumed in the same place in which they are produced. The geographical separation of production and consumption leads to a shift in energy consumption and associated environmental pressures among countries. This shift has been convincingly demonstrated with regard to energy use, land use, greenhouse gases and other ecological issues [1–5]. Similarly, local air pollution problems associated with demands for products and services from other countries should be given sufficient attention.

Among the pollutants highlighted for adverse effect on human health, fine particulate matter (PM_2.5_) has been given particular attention [6–8]. Ambient PM_2.5_ is contributed by primary emissions from coal combustion, diesel vehicles and industrial processes, as well as the secondary sources from oxidation of precursors such as sulfur dioxide (SO_2_), nitrogen oxides (NO*_x_*) and volatile organic compounds (VOCs) [9]. Although secondary aerosols account for a large fraction of ambient PM_2.5_, their production is strongly dependent on the nonlinear chemistry of atmospheric oxidants, making it difficult to precisely trace the origin of PM_2.5_ [10,11]. In addition, oxidation of gas-phase pollutants is on a timescale from hours to days, leading to a larger spatial mismatch between the precursors' emission site and the formation of secondary PM_2.5_ aerosols, when compared with primary PM_2.5_ [12–14]. Hence, in this study, we focus only on the primary PM_2.5_ emissions.

An assessment of the impact of primary PM_2.5_ on human health and the environment must begin with emission quantification [15–19]. Global anthropogenic primary PM_2.5_ emissions were estimated at 40 Tg in 2007, of which 87.7% originated in developing countries such as China [15]. In the past 50 years, PM_2.5_ emissions from developed countries have decreased dramatically owing to the implementation of a variety of air quality improvement regulations [20,21], whereas in certain developing countries, such as China and India, primary PM_2.5_ emissions have increased substantially owing to the rapid expansion of coal-based industries. Economic growth and rapid industrialization are clearly the drivers of PM_2.5_ emissions in emerging markets [22]. However, it has been found that export of products or services is the only final demand category that drives primary PM_2.5_ emission growth between 1997 and 2010 in China [23,24]. Linking the consumers to the producers may, therefore, provide us valuable insights into effectively mitigation.

A traditional bottom-up inventory accounts for PM_2.5_ emissions directly released within territorial boundaries and based on activity rates and technological data [15,17,18]. These inventories attribute the emissions to where they were generated, without considering the place in which the related commodities were ultimately consumed [25]. Production-based accounting is an essential input for global atmospheric models that can assess related local and downwind air quality and climate impact [26,27]; this type of accounting allows for mitigation of end-of-pipe emissions in the production of commodities, but fails to trace the final consumers of the commodities, which ultimately initiate the production [28]. Given this shortcoming, the ‘consumption-based accounting’ (also referred to as ‘footprint’ calculation) [29] is used in this study to trace PM_2.5_ emissions to the final consumer of the applicable commodities [2,30–32]. Tele-connection is a concept from atmospheric sciences relating the climate phenomena to each other at large distances [33]. Recently, this idea has been used to represent the link between production and consumption in diverse location [34–36]. Consumption-based measurement permits tele-connecting both the direct and indirect emissions generated throughout the worldwide supply chain to the goods or services consumed [23,37,38]. It has been used to investigating the environmental issues such as energy consumption, [39] CO_2_ emissions [2], material consumption [40], biodiversity [3], mercury emissions [41,42], nitrogen pollution [43], water [44,45] and land use [34,46]. Consumption-based emissions of China [28,38] and emissions embodied in China's interprovincial and international trade have been calculated [23,24,47–49]. Takahashi *et al.* [50] also quantified the consumption-based PM_2.5_ carbonaceous aerosols in nine Asian countries and regions. However, analyses cover the global supply chain network and a spatially explicit consumption-based primary PM_2.5_ assessment for the entire world covering the global supply chain do not exist.

This study traces the flow of primary PM_2.5_ emissions along international trade from original sources of pollution to the regions of final consumption, using a global trade database for 134 countries and 57 economic sectors [51]. The objective of this study is to understand the final consumption of products related to the local primary PM_2.5_ emissions from the perspective of consumption specifically with regard to where and for what purpose the related products are consumed. In addition, by quantifying the emissions transferred by international trade, this study suggests complementary policy insights for the future development of the LRTAP convention and other frameworks whose objective is cross-boundary pollution control.

## 2. Material and methods

### (a) Multi-region input–output analysis

Multi-region input–output (MRIO) analysis is emerging as a means of linking final demand with the associated environmental pressure across the world—as set against the backdrop of globalization and recent interest in a life cycle perspective [29,52,53]. In this framework, we first conducted a production-based PM_2.5_ emission inventory (*F*_Pr_) using 134 countries/regions (electronic supplementary material, table S1) and 57 industry sectors (electronic supplementary material, table S2) on the basis of previous studies [15,54]. Thereafter, by allocating the direct and indirect PM_2.5_ emissions to final consumer demand, we derived a global consumption-based PM_2.5_ emission inventory for 2007. The PM_2.5_ emission intensities for each of the 134 countries and 57 industry sectors were also calculated. These results illustrate the differences in PM_2.5_ emissions (direct and indirect) embodied in one unit of product [55,56]. Notably, we traced all the emissions associated with consumed goods back to the original source of the emissions even when the products were transferred through other countries/regions or were intermediate constituents in a multi-regional supply chain. In this vein, we also quantified the virtual PM_2.5_ emission flow throughout international trade in primary and manufactured products and services. The difference between production- and consumption-based emissions represent the net effect of emissions embodied in trade, which equals emissions embodied in exports minus emissions embodied in imports. A positive value indicates the net export of PM_2.5_ emissions, whereas a negative value indicates the net import of emissions.

The MRIO table is essential for investigations into final consumption attributions of emissions caused by national final demands for products and services [2,32,57–59] and can identify the virtual flow of emissions throughout international trade [4,60]. We constructed a fully coupled MRIO table in 2007 based on the Global Trade Analysis Project (GTAP) database [61]. The MRIO table covers the entire economic structure—including multiple regions (most regions in the present analysis are individual countries, as described in electronic supplementary material, table S1), multiple sectors and monetary flows between industrial sectors and regions [61,62].

For the global economy with *M* regions and *N* industries in each region, $z_{ij}^{rs}$ (*r*, *s* = 1, 2, … *M*; *i, j* = 1, 2 … *N*) represents an intermediate product sold from industry *i* in country *r* to industry *j* in country *s*. $y_{i}^{rs}$ (*r, s* = 1, 2, … *M*; *i* = 1, 2 … *N*) represents the finished goods (opposite to raw materials and intermediate goods) sold from industry *i* in country *r* to final consumers in country *s*. $x_{i}^{r}$ is the total output of industry *i* in country *r*. As a result, for industry *i* in country *r*, we have

2.1 $$x_{i}^{r}=\sum_{s=1}^{M}\sum_{j=1}^{N}z_{ij}^{rs}+\sum_{s=1}^{M}y_{i}^{rs}.$$

A technical coefficient $a_{ij}^{rs}=z_{ij}^{rs}\text{/}x_{j}^{s}$ is defined as the proportion of input (in monetary unit) from sector *i* in region *r* to produce one unit of output from sector *j* in region *s*. Then, equation (2.1) can be formulated as

2.2 X=AX+Y,

where *X* = ($x_{i}^{r}$), *A* = ($a_{ij}^{rs}$), and *Y* = ($y_{i}^{rs}$).

By incorporating a vector of emission intensity, a *T* × *T* emission multiplier matrix *E* can be calculated as (*T* *=* *M* *×* *N*)

2.3 $$E=\hat{h}{\left(1-A\right)}^{-1}=\hat{h}L,$$

where $\hat{h}$ is a diagonal vector representing sector-specific PM_2.5_ emissions per unit of economic output, which is defined as the direct emission intensity; *I* is the identity matrix and *A* is the matrix shown in equation (2.2). *L* is the Leontief matrix which captures both direct and indirect economic inputs to produce one unit of final demand in monetary value; similarly, an element $E_{c,d}^{r,s}$ (*r, s* *=* 1, 2, … *M*; *c, d* = 1, 2, … *T*) in matrix *E* is the emissions in sector *c* of region *r* instigated by the unit final demand in sector *d* of region *s*. Any column vector of the matrix *E,* $E_{:,d}^{:,s}$ (denoting all sectors in column (*s − *1) * T *+* *d*) measures emissions from all sectors that have been embodied in the unit final consumption of goods in sector *d* of region *s* and is referred to as the embodied emission intensity (EEI) of sector *d* in region *s.* The $E_{:,d}^{:,s}$ along the global supply chain also follows

2.4 $$\begin{array}{rl} E_{:,d}^{:,s} & =\left[e_{1}^{1}\partial_{1,d}^{1,s}+e_{2}^{1}\partial_{2,d}^{1,}+\cdots +e_{i}^{1}\partial_{N,d}^{1,s}\right]+\left[e_{1}^{2}\partial_{1,d}^{2,s}+e_{2}^{2}\partial_{2,d}^{2,s}+\cdots +e_{i}^{2}\partial_{N,d}^{2,s}\right]+\cdots \\ & \;+\left[e_{1}^{M}\partial_{1,d}^{M,s}+e_{2}^{M}\partial_{2,d}^{M,s}+\cdots +e_{i}^{M}\partial_{N,d}^{M,s}\right] \end{array}$$

where $e_{i}^{r}$ is the element of $\hat{h}$ (sector *i* in region *r*), $\partial_{c,d}^{r,s}$ is the element of *L* (row (*r −* 1)* × N* *+* *c* and column (*s* *−* 1)* × N* *+* *d*), representing inputs required for sector *d* in region *s* from sector *c* in region *r.* In equation (2.4), when *r* = *s*, $\left[e_{1}^{r}\partial_{1,d}^{r,s}+e_{2}^{r}\partial_{2,d}^{r,s}+\cdots +e_{i}^{r}\partial_{N,d}^{r,s}\right]$ is the emissions generated domestically owing to unit final demand in sector *d* in region *s*, the remaining part representing the emissions generated outside region *s*. Therefore, each EEI links to emissions both domestically and outside the region.

The fundamental principle used to assess the PM_2.5_ emissions embodied in final goods is to multiply the EEI by the final demand in each sector. The PM_2.5_ emissions associated with final consumption in region *r* (*F_Cr_*) may be calculated as

2.5 $$F^{r}=h{\left(I-A\right)}^{-1}Y^{\cdot r}=EY^{\cdot r},$$

where *h* is a row vector represents direct emission intensity, $Y^{\cdot r}={\left(Y^{1r}\;Y^{2r}\;Y^{3r}\;\cdots \;Y^{Mr}\right)}^{T},$ is the final demand vector of region *r*. *Y^rr^* is the domestic final consumption of region *r.* The MRIO model endogenously calculates not only the domestic output, but also the output in all other regions resulting from international trade in intermediate products.

In addition, consumption-based emissions ($F^{r}{\;}^{*}$) with inclusion of direct residential emissions (*Fh*) in the region *r* can be calculated as

2.6 $$F^{r}{\;}^{*}=EY^{\cdot r}+Fh.$$

From this framework, PM_2.5_ emission transfers from region *r* to region *s* are calculated as

2.7 $$F^{sr}={\tilde{h}}^{s}{\left(I-A\right)}^{-1}Y^{\cdot r}=EY^{\cdot r},$$

when *r* = *s*, *F*^*rr*^ is a vector with its elements representing emissions related to final consumption produced locally; when *r *≠ *s*, *F*^*rs*^ denotes emissions released in region *r* related to cross-regional final products consumed in region *s*. ${\tilde{h}}^{s}$ is a vector of the corresponding direct emission intensity for region s but zero for all other regions.

### (b) Production-based PM_2.5_ emission inventory

The global production-based PM_2.5_ emission inventory was derived from Peking University's PM_2.5_ Inventory for 2007 (PKU-PM-2007) [15] (http://inventory.pku.edu.cn/home.html). PKU-PM-2007 is a bottom-up emission inventory with high spatial resolution (0.1° × 0.1°) that was built on the basis of a global fuel combustion dataset (PKU-FUEL-2007, covering 64 fuel combustion processes and 14 industrial processes in 233 countries/territories) and updated PM_2.5_ emission factors [15,54]. In this study, the production-based industrial PM_2.5_ emissions (including both fossil fuel combustion and industrial processes) were based on the PKU-PM-2007 inventory, but converted into 57 sectors and 134 regions according to the country and sector information in v. 8.1 of the GTAP (see details in electronic supplementary material). Previous studies generally built global primary PM_2.5_ emission inventories with high spatial resolution, we first built the economic sector-based emission inventory from both the production and consumption perspectives. Energy consumption by sector, GDP and population data of each region were all derived from the GTAP database. Further details to the development of PKU-PM-2007 and comparison with other inventories can be found in our previous studies [15,54,63].

## 3. Results

### (a) Consumption-based PM_2.5_ emissions

The sources of primary PM_2.5_ emissions in this study consist of industrial primary PM_2.5_ emission sources directly emitted from energy combustion and industrial processes, as well as direct residential emissions (e.g. cooking and heating). We use ‘industrial PM_2.5_ emissions’ to describe emissions from all economic sectors (i.e. agriculture, industrial activity, power generation, transportation and non-transportation services). This classification is different from what has been used in previous studies that divide PM_2.5_ emissions into four categories: industrial, power, transportation and residential [15,64,65]. This study focuses on primary industrial emissions and primary residential emissions that amount to 24 and 15.6 Tg, respectively.

### (b) Regional PM_2.5_ emissions

**Figure 1.**
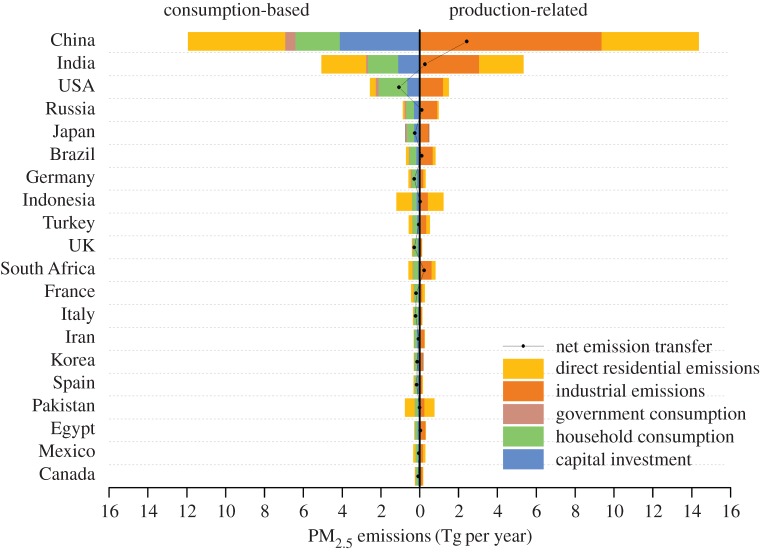
Production-based (industrial emissions and direct residential emissions) versus consumption-based (government consumption, household consumption, capital investment and direct residential emissions) PM_2.5_ emissions in 2007 in different countries. The difference between production-based emissions and consumption-based emissions represents the net emission transfer via trade and a positive value indicate that emissions embodied in exports are less than that in imports and thus a net importer of emissions. (Online version in colour.)

**Figure 2.**
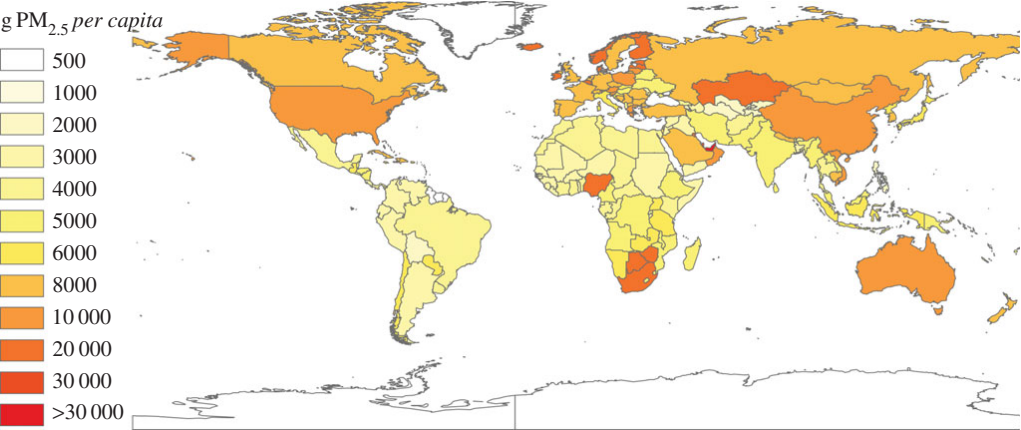
*Per capita* consumption-based emissions (g PM_2.5_ *per capita*). (Online version in colour.)

Production-based PM_2.5_ emissions denote emissions resulting from fuel combustion, industrial processes and residential life, mainly generated in China (14.4 Tg), India (5.3 Tg), the USA (1.5 Tg), Indonesia (1.2 Tg) and Russia (0.98 Tg) (figure 1). However, from a consumption perspective, the top PM_2.5_ emitters were still among the world's largest economies, i.e. China (11.9 Tg), India (5.1 Tg), the USA (2.6 Tg), Indonesia (1.2 Tg) and Russia (0.86 Tg; detailed information is provided in electronic supplementary material, figure S1 and table S3). These five countries also occupied the top five positions for consumption-based CO_2_ emissions in 2004 [2]. Notably, the pattern of consumption-based emission for PM_2.5_ is different from that of CO_2_, which the consumption-based CO_2_ emission has been widely studied by previous literature. Based on global production-based emission inventory [15] and MRIO table derived from GTAP database [66], China's consumption-based PM_2.5_ emissions is found to more than four times the USA's in 2007, whereas China's consumption-based CO_2_ emissions amounted to only 61% of those of the USA in the same year [2]. This difference stemmed from the larger difference in emission factor, sectoral emission intensity and substantial residential emissions across regions. On a *per capita* basis, high *per capita* consumption-based emissions in some developed countries were highlighted (e.g. 16.2 and 13.1 kg person^−1^ in Luxembourg and Finland, respectively; figure 2). Conversely, *per capita* consumption-based emissions are lowest in the regions characterized with less developed economy, mainly in Africa and Asia (e.g. Kyrgyzstan, Guinea). This distribution pattern is very similar to *per capita* consumption-based CO_2_ emissions and can be attributed to the remarkable disparity in living standards between countries [2].

From the sectors in terms of consumption-based industrial emissions, final demand by consumers consisted of three categories (i.e. household consumption, government consumption and investment), which contributed 51, 7 and 42% of global PM_2.5_ emissions, respectively. The composition of final demand was closely linked to income and associated expenditure patterns and lifestyles [67]. Figure 1 shows the composition of consumption-based PM_2.5_ emissions in the top 20 countries, which were responsible for approximately 80% of the global total. In developing countries, a significant portion of PM_2.5_ emissions (e.g. 60% in China and 40% in India) were related to capital investment (particularly in construction) [23,38], suggesting that investment contributed to an even greater proportion of PM_2.5_ emissions than the proportion in their GDPs. Construction drives PM_2.5_ emissions by creating an increasing market demand for the large-scale production expansion of cement, steel and other emission-intensive material, which has much larger emission intensity than the same products in other developed regions (more details in the following sections). Until the vast difference between the emission intensities of Chinese industries and that in developed regions is reduced, PM_2.5_ emissions in China is difficult to cut down. However, household consumption (i.e. demands by households for finished goods and services) was the dominant contributor for developed countries (e.g. the USA (65%), Brazil (62%), Western Europe (61% in total), Russia (49%) and Japan (50%)). Particularly, a substantial portion of USA and Western Europe's PM_2.5_ emissions associated with household consumption, i.e. 33 and 26%, respectively, were embodied in non-transportation services (i.e. trade, insurance, dwelling, see details in electronic supplementary material, table S2).

In summary, for developing countries, rationally controlling the expansion of construction and shifting investment to tertiary industries (e.g. technological innovation), as well as lowering emission intensity (such as improving the energy mix) and tightening environmental standards will yield benefits that lower PM_2.5_ emissions [68]. In contrast, for developed countries, promoting green consumption is a viable approach to reducing PM_2.5_ emissions.

### (c) PM_2.5_ emissions for industrial sectors

**Figure 3.**
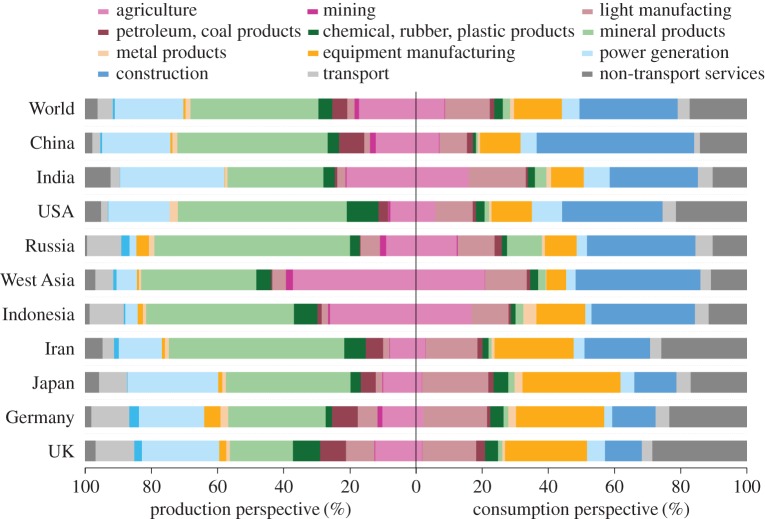
Comparison of sectoral contribution to production-based and the consumption-based PM_2.5_ emissions for selected countries. (Online version in colour.)

Figure 3 presents a comparison of sectoral PM_2.5_ emissions from both the production and consumption perspectives for the selected regions. The sectoral contribution differs significantly between the two perspectives and across the regions. From a production perspective, global PM_2.5_ emissions derived mainly from mineral products (38.5%), power generation (20.6%) and agriculture (17%). By allocating global primary PM_2.5_ emissions to countries and industrial sectors according to the final demand of consumers for finished goods, the consumption pattern indicated that 29.7, 11.9 and 9.6% of total industrial PM_2.5_ emissions are driven by the final demand for products in construction, service and machinery and equipment sectors, respectively. Notably, construction generally contributed a higher proportion of PM_2.5_ emissions in developing countries (24–37%), whereas services were responsible for higher emissions in developed countries. In general, developing countries are undergoing rapid urbanization, which requires large amounts of cement, steel and electricity and, therefore, considerable energy consumption. In addition to construction, services also contributed substantial PM_2.5_ emissions in both developing and developed countries (such as China and the USA, respectively). Although services release minimal emissions onsite, they require electricity, transport and other emission-intensive products and services as inputs along the entire supply chain, resulting in substantial indirect PM_2.5_ emissions [69].

### (d) Embodied PM_2.5_ intensity in industrial sectors

**Figure 4.**
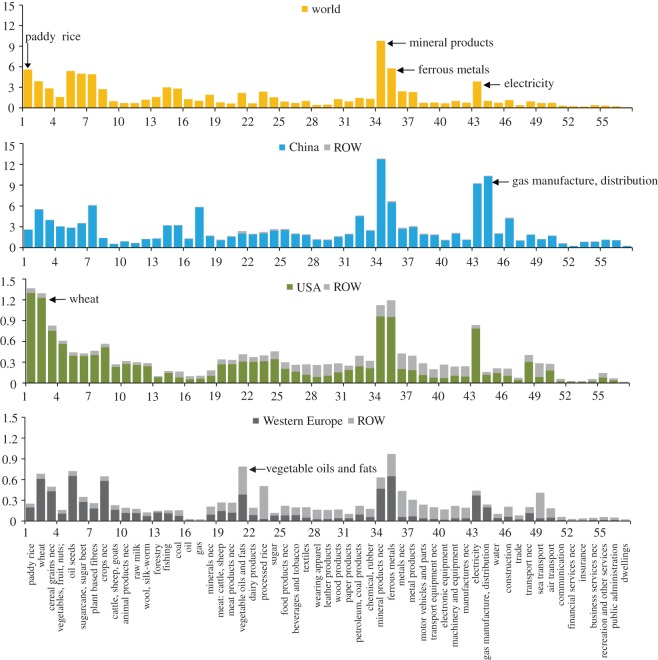
Mean embodied PM_2.5_ emission intensity (g per $) for 57 sectors (electronic supplementary material, table S2) of the world, China the USA and Western Europe. Embodied Intensity includes contributions from domestic and rest of the world (ROW) supply chains. (Online version in colour.)

Emission intensity is frequently used as an indicator to represent the technology level. The direct and indirect PM_2.5_ emissions related to final demand for goods or services in each particular industry are referred to as ‘EEI’ [55,56]. According to equation (2.4), such EEI reflects energy intensity (energy consumed per dollar of final demand), emission factor (PM_2.5_ emissions per unit of energy consumed) and trade structure of intermediate goods. Average EEIs varied considerably across sectors (figure 4), from 0.09 g per $ in dwellings to 9.8 g per $ in mineral products. Moreover, a large disparity in EEIs was observed for the same sector in different regions. Figure 4 depicts the mean EEIs of 57 sectors in China, the USA and Western Europe. The EEIs in China were approximately two to 10 times higher than the corresponding EEIs in the USA and Western Europe because of the high emission intensity of China's domestic supply chain. Western European countries usually have much lower domestic EEIs than other countries, mainly because of the high proportion of nuclear and hydropower (e.g. France and Sweden) and advanced clean production technologies. In contrast, 75% of China's primary energy source was supplied by coal in 2007, the highest level among major energy-consuming countries [68]. Emissions in power sector are found to enhance almost all sectors' EEI in China, because more than 80% of the economic sectors in China follow a similar manner in requiring the material or electricity input in their supply chain [37]. The high EEI in China was also underpinned by the low production efficiency and low environmental standards compared with developed regions. Previous study has shown that the life cycle CO_2_ emissions per unit mass of each product (similar to the EEI) for Chinese products was on average 4.4 times higher than the same products made in Europe [68]. The emission factor of PM_2.5_ is more dependent on technology and end-of-pipe control measures, thus the difference of embodied emission intensities across regions is much larger than CO_2_.

Moreover, owing to the great differences in emission intensity across different countries, importing/exporting commodities exerts influences beyond the domestic scale. For instance, the EEI of machinery and equipment was 2.11 g per $ in China (1.98 domestically and 0.13 abroad) and 0.24 g per $ in the USA (0.11 domestically and 0.13 abroad). If producing one piece of equipment requires a 10 000 dollar input, then 2.4 kg PM_2.5_ (1.1 kg domestically and 1.3 kg abroad) was released if it was produced within the USA as opposed to 21.1 kg (19.8 kg domestically and 1.3 kg abroad) in China. Thus, international trade may significantly change the magnitude and distribution of global PM_2.5_ emissions.

### (e) PM_2.5_ emissions embodied in trade

**Figure 5.**
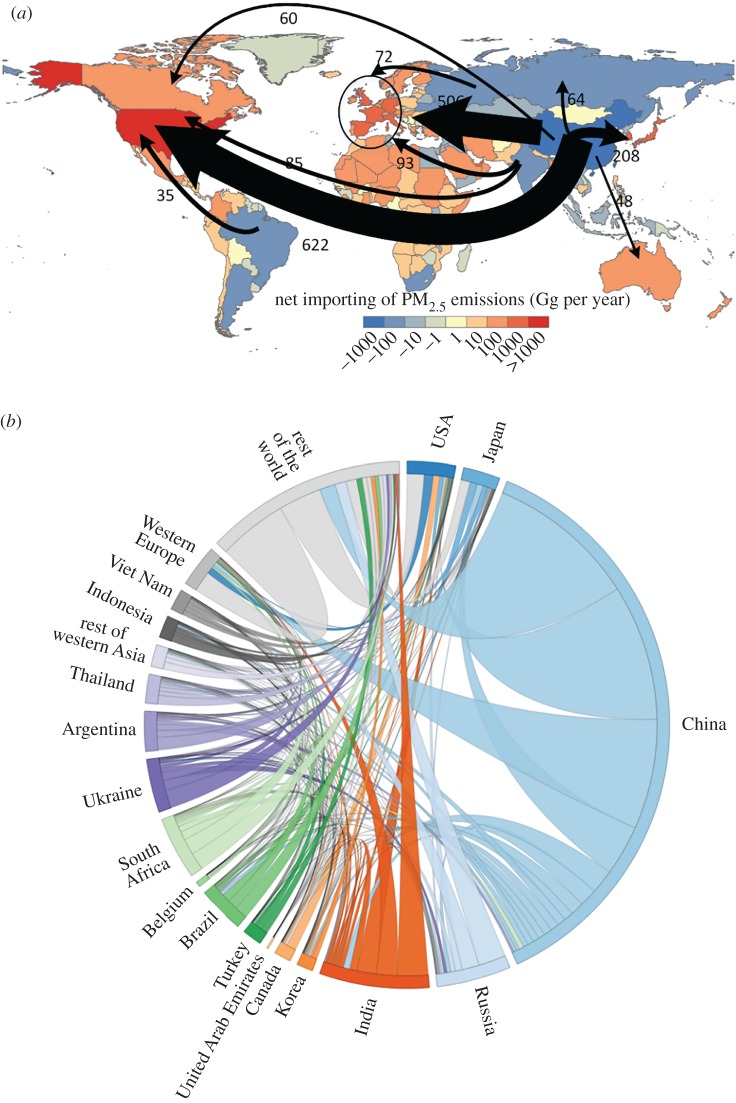
Production-based PM_2.5_ emissions linked to foreign consumption. (*a*) The largest interregional flow of emissions embodied in trade (Gg per year) from dominant net exporting countries (blue) to the dominant importing countries (red). Flows to and from Western Europe are aggregated and include the United Kingdom, Germany, France, Switzerland, Italy, Spain, Sweden, Luxembourg and the Netherlands; (*b*) the interregional flow pattern of PM_2.5_ emissions embodied in the global trade system (Gg per year) among 20 aggregated regions. The flow from each region represents PM_2.5_ emissions embodied in exported commodities to the other end of the link, and the link colour corresponds to the regions with greater exports. (Online version in colour.)

Globally, nearly 30% of PM_2.5_ emissions (7.2 Tg) were linked to the production of goods or services consumed outside the boundaries of the producing country in 2007 on the basis of equation (2.6). Figure 5 further illustrates the interregional flows of PM_2.5_ embodied in trading goods and services. Note that the direction of the virtual PM_2.5_ flow is the same with the flow of the commodities, as previous studies did [2,70]. Certain developed countries, including the USA (1058 Gg), Japan (248 Gg), Germany (278 Gg), the United Kingdom (272 Gg), Italy (203 Gg) and France (22 Gg), had the measurable PM_2.5_ emissions embodied in imports, which contributed 40–70% to their consumption-based PM_2.5_ emissions (figure 1). By contrast, countries such as China (6948 Gg), India (2782 Gg), South Africa (240 Gg), Ukraine (191 Gg), and Russia (116 Gg) exported 10–115% (India–Ukraine) of their total consumption-based PM_2.5_ emissions via exporting commodities in 2007.

Figure 5*a* also shows the key interregional links from the largest net importing countries (red) to the largest net exporting countries (blue). The dominant pattern is that 27, 29 and 26% of consumption-based PM_2.5_ emissions in the USA, Japan and Western Europe, respectively, were linked to emissions in the production of exports in China in 2007. Notably, the USA imported more embodied PM_2.5_ emissions than any other country by importing products from China (622 Gg), India (85 Gg) and Brazil (35 Gg). By contrast, approximately 30% or 2.7 Tg of PM_2.5_ emissions in China were linked to consumption in foreign countries/regions, which accounts for 38% of the global total of traded emissions (7.2 Tg). The emissions imported by developed countries from emerging countries reinforced the already large global disparity in the spatial heterogeneity of PM_2.5_ emissions.

The consumers in one country typically tele-connect PM_2.5_ emissions in foreign countries by importing a wide variety of goods and services for final consumption purposes. Further exploration of the commodity content of trade activities (electronic supplementary material, figure S2) shows that PM_2.5_ emissions embodied in the USA imports exceed those of any other country or region and were primarily embodied in machinery and equipment (131 Gg), unclassified manufactured goods (43 Gg), clothing (36 Gg), mineral products (23 Gg), leather products (23 Gg) and intermediate goods (875 Gg). These imports were offset by additional emissions in USA exports of machinery and equipment (18 Gg), agricultural products (11 Gg) and intermediate goods (148 Gg). The patterns were similar in Japan and Western Europe (UK, Germany, France, Switzerland, Italy, Spain, Sweden, Luxembourg and the Netherlands) with substantial emissions embodied in imports to meet demand for machinery and equipment, clothing, food products and chemical, rubber and plastic products.

**Figure 6.**
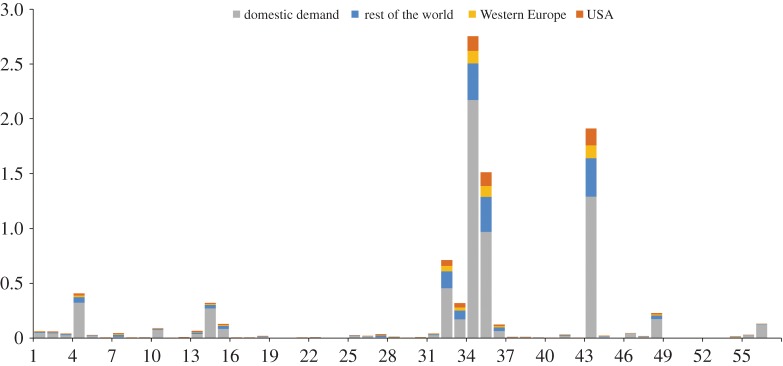
Sectoral PM_2.5_ emission in China related to domestic demand (grey) and the demand from Western Europe (yellow), the USA (orange) and the rest of the world (blue). (Online version in colour.)

Net exporters of emissions—such as China—with vast exports tended to generate much domestic PM_2.5_ emissions for trade purposes. The emissions from production of exports from China were mainly caused by exporting commodities such as machinery and equipment, unclassified manufactured goods, clothing, mineral products and intermediate goods (electronic supplementary material, figure S2), which drove PM_2.5_ emissions generated from the production of mineral products (1126 Gg), power generation (624 Gg), agriculture (277 Gg) and petroleum and coal products (258 Gg). Figure 6 shows China's sectoral PM_2.5_ emissions, which were divided into four categories based on final demand. The top three sectors—mineral products (2.5 Tg), electricity (1.9 Tg) and ferrous metals (1.5 Tg)—together contributed two-thirds of China's total PM_2.5_ emissions; 21, 32 and 36%, respectively, were instigated by demand from foreign countries (figure 6). More importantly, the embodied emission intensities of products in these three sectors in China were more than 10-fold of the same products made in Europe and USA. There is a considerable opportunity to reduce primary PM_2.5_ emissions in China by focusing on these sectors where more energy efficiency or end-of-pipe technologies can be installed and shifting the energy mix towards cleaner energy sources. Such improvements can be supported by both domestic and international efforts to facilitate technology transfer into these critical sectors. Stricter regulation, particularly in these sectors, is also crucial in reducing primary PM_2.5_ emissions. Furthermore, in nine of the 57 sectors, emissions resulting from domestic demand were even smaller than those from foreign demand. In particular, nearly three-quarters of the emissions from the production of electronic equipment, textiles and plant-based fibres were emitted during the production of export-related products. Similarly, China's stark imbalance of CO_2_ emissions embodied in trade was also embodied in exports of machinery and equipment, clothing and textiles and substantial exports of intermediate goods [71]. As the production process requires vast amounts of energy and inputs from other sectors, the results drive both CO_2_ and PM_2.5_ emissions in sectors such as electricity and ferrous metals.

## 4. Discussion

International policies to address transboundary air pollution are still in development, such as the convention on long-range transboundary air pollution (LRTAP) and the task force on hemispheric transport of air pollution (TF HTAP), despite the significant role of international trade. The TF HTAP is organized under the auspices of the UNECE LRTAP convention and aims to understand how emissions in an outside region affect the air quality, health and climate in a given region [72]. However, these policies are limited by identification of only physical transport [73]. These indirect, trade-induced primary emissions efficiently elevate PM_2.5_ concentrations in the receptor regions, which are 20–200 times larger than the concentration enhancement resulting from intercontinental transport, particularly between North American (NA) and European (EU) sources and East Asian (EA) and South Asian (SA) receptors (table 1; electronic supplementary material, table S4 and TOC Art) [26,27]. Even for a much smaller region (i.e. China), trade-induced intraregional transfer of emissions is also found to be more important than atmospheric transport of pollution from one province to another [47]. It is therefore clear that a furthering of the understanding of both the international atmospheric transport and virtual transport could improve science cooperation at the international scale and work towards mitigation of relevant emission sources [24,47,50,74].

**Table 1.** Virtual PM_2.5_ emissions flow from a net importer of products (source region of PM_2.5_) to a net exporter of products (receptor). The contributions of a region to itself (referred to ‘domestic’) are shown in italics (unit: Gg).

	receptor region			
source region	NA	EU	SA	EA
NA	*1343.2*	55.0	100.8	800.7
EU	74.1	*1008.5*	126.0	716.7
SA	6.6	7.2	*2577.5*	82.2
EA	57.6	29.9	59.2	*7666.5*

The analysis of consumption-based emission enables the evaluation of emission transfers between countries [2,4] and demand-side policy options [75], and facilitates the exchanges of information between the producers and final consumers. Developed regions with more emissions embodied in imports and mature technical capabilities could provide other developing regions with assistance on emissions estimation, monitoring methodology, modelling techniques, management and control programme implementation, and enforcement. Developing regions, in return, could provide better information about the sources, character and reduce emissions attributed to developed regions' consumption. Given the rapid growth of international trade [76,77], such policies must also have a global coverage and reach. To achieve it, comprehensive information on the global primary PM_2.5_ transfers from original emission sources to regions of consumption must be available, and this is the main contribution of our study. The consumption-based primary PM_2.5_ accounting shares concepts and methods with consumption-based CO_2_ [2], material [40], biodiversity [3], mercury [41,78], nitrogen pollution [43] and water [44,45]. Therefore, debates about countries' responsibility for the environmental impact of their imports apply equally to the primary PM_2.5_: consumption-based PM_2.5_ policies can enforce controls on PM_2.5_ leakage and trade regulation, just as demand-side abatement of carbon emissions, but it would also face different obstacles in world trade agreements due to primary PM_2.5_'s short atmospheric lifetime.

Yet, the appeal of the consumption-based accounting is that it enables consumers to create competition among producers in a race to the top, particularly in the current globalized world with far-reaching supply chains. More environmentally aware consumers might also push firms to differentiate their domestic and international suppliers on the basis of EEIs. In addition to regulating local emissions and adopting PM_2.5_ removal technologies, additional efforts might also be made by consuming countries to weigh EEIs more heavily in selecting their key trading partners, with the goal of obtaining lower overall consumption-based emissions and reducing the international pollution disparity from globalized consumption.

The uncertainties in the consumption-based emission inventory arise mainly from the biases when compiling the production-based PM_2.5_ emission inventory [15], and the uncertainties associated in the MRIO table. A detailed description of the major uncertainties in production-based PM_2.5_ emission inventory has been given by Huang *et al.* [15]. Basically, biases in emission factors and technology split could significantly enlarge the uncertainty range of emission inventory. For the MRIO table, sector aggregation may cause substantial uncertainties in consumption-based accounting [61]. However, the GTAP database includes 57 sectors, and can provide sufficient details to link PM_2.5_ emissions to each individual economic sector [79]. Consequently, the GTAP database has been widely used in the environmental MRIO analyses [4,29,71,80]. Therefore, even with those uncertainties, the results in this study may provide valuable insights into PM_2.5_ emissions management. In addition, the redistribution of PM_2.5_ emissions may cause increased/decreased morbidity or mortality associated with PM_2.5_ exposure, which highly relates to the spatio-temporal distribution of PM_2.5_ concentrations and population density [50,81]. In our follow-up studies, we extend the framework developed here and use the three-dimensional chemical transport model in conjunction with the tracers tagging technique to explicitly compare the health impacts of PM_2.5_ exposure caused by atmospheric movement and virtual transport via trade [47]. In addition, the precursors (such as VOCs, SO*_x_*, NH_3_ and NO*_x_*) can be incorporated into a chemical transport model to evaluate the contribution of secondary aerosols.

## Supplementary Material

Supporting tables and figures

## Data accessibility

All data is available as supplementary material.

## References

[1] Wei T et al. 2012 Developed and developing world responsibilities for historical climate change and CO_2_ mitigation. Proc. Natl Acad. Sci. USA 109, 12 911–12 915. (doi:10.1073/pnas.1203282109) 10.1073/pnas.1203282109 PMC3420160 22826257

[2] Davis SJ, Caldeira K 2010 Consumption-based accounting of CO_2_ emissions. Proc. Natl Acad. Sci. USA 107, 5687–5692. (doi:10.1073/pnas.0906974107) 20212122 10.1073/pnas.0906974107 PMC2851800

[3] Lenzen M, Moran D, Kanemoto K, Foran B, Lobefaro L, Geschke A 2012 International trade drives biodiversity threats in developing nations. Nature 486, 109–112. (doi:10.1038/nature11145) 22678290 10.1038/nature11145

[4] Peters GP, Hertwich EG 2008 CO_2_ embodied in international trade with implications for global climate policy. Environ. Sci. Technol. 42, 1401–1407. (doi:10.1021/es072023k) 18441780 10.1021/es072023k

[5] Steen-Olsen K, Weinzettel J, Cranston G, Ercin AE, Hertwich EG 2012 Carbon, land, and water footprint accounts for the European Union: consumption, production, and displacements through international trade. Environ. Sci. Technol. 46, 10 883–10 891. (doi:10.1021/es301949t) 23013466 10.1021/es301949t

[6] Apte JS, Marshall JD, Cohen AJ, Brauer M 2015 Addressing global mortality from ambient PM2. 5. Environ. Sci. Technol. 49, 8057–8066. (doi:10.1021/acs.est.5b01236) 26077815 10.1021/acs.est.5b01236

[7] Lelieveld J, Evans J, Fnais M, Giannadaki D, Pozzer A 2015 The contribution of outdoor air pollution sources to premature mortality on a global scale. Nature 525, 367–371. (doi:10.1038/nature15371). 26381985 10.1038/nature15371

[8] West J et al. 2016 What we breathe impacts our health: improving understanding of the link between air pollution and health. Environ. Sci. Technol. 50, 4895–4904. (doi:10.1021/acs.est.5b03827) 27010639 10.1021/acs.est.5b03827 PMC12303222

[9] Megaritis A, Fountoukis C, Charalampidis P, Pilinis C, Pandis SN 2013 Response of fine particulate matter concentrations to changes of emissions and temperature in Europe. Atmos. Chem. Phys. 13, 3423–3443. (doi:10.5194/acp-13-3423-2013)

[10] Kolb CE, Worsnop DR 2012 Chemistry and composition of atmospheric aerosol particles. Annu. Rev. Phys. Chem. 63, 471–491. (doi:10.1146/annurev-physchem-032511-143706) 22404591 10.1146/annurev-physchem-032511-143706

[11] Jacob D 1999 Introduction to atmospheric chemistry. Princeton, NJ: Princeton University Press.

[12] Liu X et al. 2013 Formation and evolution mechanism of regional haze: a case study in the megacity Beijing, China. Atmos. Chem. Phys. 13, 4501–4514. (doi:10.5194/acpd-12-16259-2012)

[13] He H et al. 2014 Mineral dust and NO*_x_* promote the conversion of SO_2_ to sulfate in heavy pollution days. Sci. Rep. 4, 4172 (doi:10.1038/srep04172) 24566871 10.1038/srep04172 PMC3933828

[14] Robinson AL, Donahue NM, Rogge WF 2006 Photochemical oxidation and changes in molecular composition of organic aerosol in the regional context. J. Geophys. Res. Atmospheres (1984–2012) 111, D03302 (doi:10.1029/2005JD006265)

[15] Huang Y et al. 2014 Quantification of global primary emissions of PM_2.5_, PM_10_, and TSP from combustion and industrial process sources. Environ. Sci. Technol. 48, 13 834–13 843. (doi:10.1021/Es503696k) 10.1021/es503696k 25347079

[16] Zhang Q et al. 2009 Asian emissions in 2006 for the NASA INTEX-B mission. Atmos. Chem. Phys. 9, 5131–5153. (doi:10.5194/acp-9-5131-2009)

[17] Janssens-Maenhout G et al. 2011 EDGAR-HTAP: a harmonized gridded air pollution emission dataset based on national inventories. European Commission. Institute for Environment and Sustainability.

[18] Van Aardeene J 2010 Emissions Database for Global Atmospheric Research. (Updated 10 June 2010). See http://edgar.jrc.ec.europa.eu/index.php (accessed 6 October 2010).

[19] Klimont Z, Cofala J, Bertok I, Amann M, Heyes C, Gyarfas F 2002 Modelling particulate emissions in Europe: a framework to estimate reduction potential and control costs, IR-02-076. Laxenburg, Austria: International Institute for Applied Systems Analysis.

[20] Domenici P 1979 Clean Air Act Amendments of 1977. Nat. Resour. J. 19, 475.

[21] Agency, U. S. E. P. 2010 History of the Clean Air Act. See http://www.epa.gov/air/caa/requirements.html.

[22] Lu Z, Zhang Q, Streets DG 2011 Sulfur dioxide and primary carbonaceous aerosol emissions in China and India, 1996–2010. Atmos. Chem. Phys. 11, 9839–9864. (doi:10.5194/acp-11-9839-2011)

[23] Guan D, Su X, Zhang Q, Peters GP, Liu Z, Lei Y, He K 2014 The socioeconomic drivers of China's primary PM_2.5_ emissions. Environ. Res. Lett. 9, 024010 (doi:10.1088/1748-9326/9/2/024010)

[24] Lin J, Pan D, Davis SJ, Zhang Q, He K, Wang C, Streets DG, Wuebbles DJ, Guan D 2014 China's international trade and air pollution in the United States. Proc. Natl Acad. Sci. USA 111, 1736–1741. (doi:10.1073/pnas.1312860111) 24449863 10.1073/pnas.1312860111 PMC3918792

[25] Peters GP 2008 From production-based to consumption-based national emission inventories. Ecol. Econ. 65, 13–23. (doi:10.1016/j.ecolecon.2007.10.014)

[26] Liu JF, Mauzerall DL, Horowitz LW, Ginoux P, Fiore AM 2009 Evaluating inter-continental transport of fine aerosols: methodology, global aerosol distribution and optical depth. Atmos. Environ. 43, 4327–4338. (doi:10.1016/j.atmosenv.2009.03.054)

[27] Liu JF, Mauzerall DL, Horowitz LW 2009 Evaluating inter-continental transport of fine aerosols: (2) Global health impact. Atmos. Environ. 43, 4339–4347. (doi:10.1016/j.atmosenv.2009.05.032)

[28] Huo H, Zhang Q, Guan DB, Su X, Zhao HY, He KB 2014 Examining air pollution in china using production- and consumption-based emissions accounting approaches. Environ. Sci. Technol. 48, 14 139–14 147. (doi:10.1021/Es503959t) 10.1021/es503959t 25401750

[29] Wiedmann T 2009 A review of recent multi-region input–output models used for consumption-based emission and resource accounting. Ecol. Econ. 69, 211–222. (doi:10.1016/j.ecolecon.2009.08.026)

[30] Hertwich EG 2005 Life cycle approaches to sustainable consumption: a critical review. Environ. Sci. Technol. 39, 4673–4684. (doi:10.1021/es0497375) 16053063 10.1021/es0497375

[31] Larsen HN, Hertwich EG 2009 The case for consumption-based accounting of greenhouse gas emissions to promote local climate action. Environ. Sci. Policy 12, 791–798. (doi:10.1021/es0497375)

[32] Hertwich EG, Peters GP 2009 Carbon footprint of nations: a global, trade-linked analysis. Environ. Sci. Technol. 43, 6414–6420. (doi:10.1021/es803496a) 19746745 10.1021/es803496a

[33] Glantz MH, Katz RW, Nicholls N 1991 Teleconnections linking worldwide climate anomalies. Cambridge, UK: Cambridge University Press.

[34] Yu Y, Feng K, Hubacek K 2013 Tele-connecting local consumption to global land use. Glob. Environ. Change 23, 1178–1186. (doi:10.1016/j.gloenvcha.2013.04.006)

[35] Friis C, Reenberg A 2010 Land grab in Africa: emerging land system drivers in a teleconnected world. Copenhagen, Denmark: GLP International Project Office, University of Copenhagen.

[36] Friis C, Nielsen JØ, Otero I, Haberl H, Niewöhner J, Hostert P 2015 From teleconnection to telecoupling: taking stock of an emerging framework in land system science. J. Land Use Sci. 1–23. (doi:10.1080/1747423X.2015.1096423)

[37] Erickson P, Allaway D, Lazarus M, Stanton EA 2012 A consumption-based GHG inventory for the US State of Oregon. Environ. Sci. Technol. 46, 3679–3686. (doi:10.1021/es203731e) 22439796 10.1021/es203731e

[38] Meng J, Liu J, Xu Y, Tao S 2015 Tracing primary PM_2.5_ emissions via Chinese supply chains. Environ. Res. Lett. 10, 054005 (doi:10.1088/1748-9326/10/5/054005)

[39] Chen ZM, Chen GQ 2013 Demand-driven energy requirement of world economy 2007: a multi-region input-output network simulation. Commun. Nonlinear Sci. 18, 1757–1774. (doi:10.1016/j.cnsns.2012.11.004)

[40] Wiedmann TO, Schandl H, Lenzen M, Moran D, Suh S, West J, Kanemoto K 2013 The material footprint of nations. Proc. Natl Acad. Sci. USA 112, 6271–6276. (doi:10.1073/pnas.1220362110) 24003158 10.1073/pnas.1220362110 PMC4443380

[41] Liang S, Wang Y, Cinnirella S, Pirrone N 2015 Atmospheric mercury footprints of nations. Environ. Sci. Technol. 49, 3566–3574. (doi:10.1021/es503977y) 25723898 10.1021/es503977y

[42] Li J 2016 An overview of mercury emissions by global fuel combustion: the impact of international trade. Renew. Sust. Energ. Rev. 41, 1167–1175. (doi:10.1016/j.rser.2014.08.073)

[43] Oita A, Malik A, Kanemoto K, Geschke A, Nishijima S, Lenzen M 2016 Substantial nitrogen pollution embedded in international trade. Nat. Geosci. 9, 111–115. (doi:10.1038/ngeo2635)

[44] Lenzen M, Moran D, Bhaduri A, Kanemoto K, Bekchanov M, Geschke A, Foran B 2013 International trade of scarce water. Ecol. Econ. 94, 78–85. (doi:10.1016/j.ecolecon.2013.06.018)

[45] Chen Z-M, Chen G 2013 Virtual water accounting for the globalized world economy: national water footprint and international virtual water trade. Ecol. Indic. 28, 142–149. (doi:10.1016/j.ecolind.2012.07.024)

[46] Weinzettel J, Hertwich EG, Peters GP, Steen-Olsen K, Galli A 2013 Affluence drives the global displacement of land use. Glob. Environ. Change 23, 433–438. (doi:10.1016/j.gloenvcha.2012.12.010)

[47] Li Y, Meng J, Liu J, Xu Y, Guan D, Tao W, Huang Y, Tao S 2016 Inter-provincial reliance for improving air quality in China: a case study on black carbon aerosol. Environ. Sci. Technol. 50, 4118–4126. (doi:10.1021/acs.est.5b05989) 26950657 10.1021/acs.est.5b05989

[48] Zhao H, Zhang Q, Guan D, Davis S, Liu Z, Huo H, Lin J, Liu W, He K 2015 Assessment of China's virtual air pollution transport embodied in trade by using a consumption-based emission inventory. Atmos. Chem. Phys. 15, 5443–5456. (doi:10.5194/acp-15-5443-2015)

[49] Meng J, Liu J, Guo S, Huang Y, Tao S 2015 The impact of domestic and foreign trade on energy-related PM emissions in Beijing. Appl. Energy (doi:10.1016/j.apenergy.2015.09.082)

[50] Takahashi K et al. 2014 Production-based emissions, consumption-based emissions and consumption-based health impacts of PM_2.5_ carbonaceous aerosols in Asia. Atmos. Environ. 97, 406–415. (doi:10.1016/j.atmosenv.2014.04.028)

[51] Andrew RM, Peters GP 2013 A multi-region input-output table based on the global trade analysis project database (Gtap-Mrio). Econ. Syst. Res. 25, 99–121. (doi:10.1080/09535314.2012.761953)

[52] Peters G, Davis S, Andrew R 2012 A synthesis of carbon in international trade. Biogeosciences 9, 3247–3276. (doi:10.5194/bg-9-3247-2012)

[53] Dietzenbacher E et al. 2013 Input–output analysis: the next 25 years. Econ. Syst. Res. 25, 369–389. (doi:10.1080/09535314.2013.846902)

[54] Wang R et al. 2013 High-resolution mapping of combustion processes and implications for CO_2_ emissions. Atmos. Chem. Phys. 13, 5189–5203. (doi:10.5194/acp-13-5189-2013)

[55] Chen G, Zhang B 2010 Greenhouse gas emissions in China 2007: inventory and input–output analysis. Energy Policy 38, 6180–6193. (doi:10.1016/j.enpol.2010.06.004)

[56] Chen Z, Chen G 2011 Embodied carbon dioxide emission at supra-national scale: a coalition analysis for G7, BRIC, and the rest of the world. Energy Policy 39, 2899–2909. (doi:10.1016/j.enpol.2011.02.068)

[57] Wiedmann T et al. 2010 A carbon footprint time series of the UK–results from a multi-region input–output model. Econ. Syst. Res. 22, 19–42. (doi:10.1080/09535311003612591)

[58] Hubacek K, Guan DB, Barrett J, Wiedmann T 2009 Environmental implications of urbanization and lifestyle change in China: ecological and water footprints. J. Clean Prod. 17, 1241–1248. (doi:10.1016/j.jclepro.2009.03.011)

[59] Guan D, Hubacek K, Weber CL, Peters GP, Reiner DM 2008 The drivers of Chinese CO(2) emissions from 1980 to 2030. Glob. Environ. Change 18, 626–634. (doi:10.1016/j.gloenvcha.2008.08.001)

[60] Guan D, Peters GP, Weber CL, Hubacek K 2009 Journey to world top emitter: an analysis of the driving forces of China's recent CO_2_ emissions surge. Geophys. Res. Lett. 36, L04709 (doi:10.1029/2008GL036540)

[61] Narayanan B, Aguiar A, McDougall R 2012 Global trade, assistance, and production: the GTAP 8 data base. West Lafayette, IN: Center for Global Trade Analysis, Purdue University, USA.

[62] Peters GP, Andrew R, Lennox J 2011 Constructing an environmentally-extended multi-regional input-output table using the GTAP database. Econ. Syst. Res. 23, 131–152. (doi:10.1080/09535314.2011.563234)

[63] Wang R et al. 2014 Exposure to ambient black carbon derived from a unique inventory and high-resolution model. Proc. Natl Acad. Sci. USA 111, 2459–2463. (doi:10.1073/pnas.1318763111) 24469822 10.1073/pnas.1318763111 PMC3932916

[64] Lei Y, Zhang Q, He KB, Streets DG 2011 Primary anthropogenic aerosol emission trends for China, 1990–2005. Atmos. Chem. Phys. 11, 931–954. (doi:10.5194/acp-11-931-2011)

[65] Lei Y, Zhang Q, Nielsen C, He K 2011 An inventory of primary air pollutants and CO_2_ emissions from cement production in China, 1990–2020. Atmos. Environ. 45, 147–154. (doi:10.1016/j.atmosenv.2010.09.034)

[66] Tukker A, Dietzenbacher E 2013 Global multiregional input–output frameworks: an introduction and outlook. Econ. Syst. Res. 25, 1–19. (doi:10.1080/09535314.2012.761179)

[67] Baiocchi G, Minx J, Hubacek K 2010 The impact of social factors and consumer behavior on carbon dioxide emissions in the United Kingdom. J. Ind. Ecol. 14, 50–72. (doi:10.1111/j.1530-9290.2009.00216.x)

[68] Poizot P, Dolhem F 2011 Clean energy new deal for a sustainable world: from non-CO_2_ generating energy sources to greener electrochemical storage devices. Energy Environ. Sci. 4, 2003–2019. (doi:10.1039/c0ee00731e)

[69] Meng J, Liu J, Guo S, Li J, Li Z, Tao S 2015 Trend and driving forces of Beijing's black carbon emissions from sectoral perspectives. J. Clean Prod. 112, 1272–1281. (doi:10.1016/j.jclepro.2015.05.027)

[70] Feng K et al. 2013 Outsourcing CO_2_ within China. Proc. Natl Acad. Sci. USA 110, 11 654–11 659. (doi:10.1073/pnas.1219918110) 10.1073/pnas.1219918110 PMC3710878 23754377

[71] Davis SJ, Peters GP, Caldeira K 2011 The supply chain of CO_2_ emissions. Proc. Natl Acad. Sci. USA 108, 18 554–18 559. (doi:10.1073/pnas.1107409108) 22006314 10.1073/pnas.1107409108 PMC3215011

[72] Mills G 2004 Mapping critical levels for vegetation. In Manual on methodologies and criteria for modelling and mapping critical loads and levels and air pollution effects, risks and trends, ch. 3, p. 52 Geneva, Switzerland: UNECE Convention on Long-range Transboundary Air Pollution.

[73] Dentener F, Keating T, orpEurope, UNECF 2010 Hemispheric transport of air pollution 2010. Part A: ozone and particulate matter, pp. 97–144.

[74] Lin J et al. 2016 Global climate forcing of aerosols embodied in international trade. Nat. Geosci. 9, 790–794. (doi:10.1038/ngeo2798)

[75] Guan DB et al. 2014 Reply to Lopez *et al*.: consumption-based accounting helps mitigate global air pollution. Proc. Natl Acad. Sci. USA 111, E2631 (doi:10.1073/pnas.1407383111) 25115000 10.1073/pnas.1407383111 PMC4084418

[76] Development, U. U. N. C. o. T. a. 2015 Key statistics and trends in international trade 2015. New York and Geneva.

[77] Development, U. U. N. C. o. T. a. 2014 UNCTAD handbook of statistics. New York and Geneva.

[78] Li JS, Chen GQ, Hayat T, Alsaedi A 2015 Mercury emissions by Beijing's fossil energy consumption: based on environmentally extended input-output analysis. Renew. Sust. Energ. Rev. 41, 1167–1175. (doi:10.1016/j.rser.2014.08.073)

[79] Su B, Huang H, Ang B, Zhou P 2010 Input–output analysis of CO_2_ emissions embodied in trade: the effects of sector aggregation. Energy Econ. 32, 166–175. (doi:10.1016/j.eneco.2009.07.010)

[80] Skelton A, Guan D, Peters GP, Crawford-Brown D 2011 Mapping flows of embodied emissions in the global production system. Environ. Sci. Technol. 45, 10 516–10 523. (doi:10.1021/Es202313e) 22050071 10.1021/es202313e

[81] Jiang X 2015 Revealing the hidden health costs embodied in Chinese exports. Environ. Sci. Technol. 49, 4381–4388. (doi:10.1021/es506121s) 25751364 10.1021/es506121s

