# Supplementary material for: Globalization and pollution: tele-connecting local primary PM2.5 emissions to global consumption
Source: Proc Math Phys Eng Sci. 2016 Nov;472(2195):20160380. doi: 10.1098/rspa.2016.0380 (PMC5134305; doi:10.1098/rspa.2016.0380)
Supplement: Supporting tables and figures [file rspa20160380supp1.docx]

Globalisation and Pollution: Tele-connecting Local Primary PM_2.5_ Emissions to Global Consumption

Jing Meng**^†^**, Junfeng Liu**^†,^** * , Yuan Xu**^‡^**, Dabo Guan**^§^**, Zhu Liu^∥^, Ye Huang**^†,⊥^**, and Shu Tao**^†^**

**^†^** Laboratory for Earth Surface Processes, College of Urban and Environmental Sciences, Peking University, Beijing, China

**^‡^** Department of Geography and Resource Management & Institute of Environment, Energy and Sustainability, the Chinese University of Hong Kong, Hong Kong, China

**^§^** School of International Development, University of East Anglia, Norwich, NR4 7TJ, UK

^∥^ Resnick Sustainability Institute, California Institute of Technology, Pasadena, California 91125, USA

**^⊥^** Laboratoire des Sciences du Climat et de l’Environnement, Commissariat à l'Energie Atomique-Centre National de la Recherche Scientifique-Université de Versailles Saint-Quentin-en- Yvelines, Centre d'Etudes Orme des Merisiers, 91191 Gif sur Yvette, France

* Corresponding author:

Email: jfliu@pku.edu.cn

Tel.:+86 (0)10 6275 7852

**Caption**

Table S1. Definition of regions.

Table S2. Definition of sectors.

Table S3. Produced, consumed and traded PM_2.5_ emissions.

Table S4. Budget of average primary fine aerosol concentrations from inter-continental transport of aerosols from each source region to each receptor region, contributions of a region on itself (referred to ‘domestic’) are shown in bold: (a) induced by production of source region [Unit: μg∙m^-3^]; (b) induced by consumption of source region [Unit: μg∙m^-3^]; (c) the ratio concentrations induced by consumption to production.

Figure S1. Distribution of consumption-based PM_2.5_ emissions (F_Cr_) in 2007 by mass of emissions in the region.

Figure S2. PM_2.5_ emissions embodied in imports and exports of the largest net importing/exporting countries/regions. Intermediate goods (gray) are those used by industries in the importing country to further processing and meet consumer demand for domestic goods.

### Supporting method

### Bridging PKU-inventory and GTAP classifications

**Table S1**. Definition of regions.

| Number | Code | Description | Member Countries |
| --- | --- | --- | --- |
| 1 | AUS | Australia | Australia, Cocos (Keeling) Islands, Christmas Island, Heard Island and McDonald Islands, Norfolk Island |
| 2 | NZL | New Zealand | New Zealand |
| 3 | XOC | Rest of Oceania | American Samoa, Cook Islands, Fiji, Micronesia Federated States of, Guam, Kiribati, Marshall Islands, Northern Mariana Islands, New Caledonia, Niue, Nauru, Palau, Papua New Guinea, French Polynesia, Solomon Islands, Tokelau, Tonga, Tuvalu, Vanuatu, Wallis and Futuna, Samoa, Pitcairn, United States Minor Outlying Islands |
| 4 | CHN | China | China |
| 5 | HKG | Hong Kong | Hong Kong |
| 6 | JPN | Japan | Japan |
| 7 | KOR | Korea Republic of | Korea Republic of |
| 8 | MNG | Mongolia | Mongolia |
| 9 | TWN | Taiwan | Taiwan |
| 10 | XEA | Rest of East Asia | Macao, Korea Democratic Peoples Republic of |
| 11 | KHM | Cambodia | Cambodia |
| 12 | IDN | Indonesia | Indonesia |
| 13 | LAO | Lao People's Democratic Republic | Lao People's Democratic Republic |
| 14 | MYS | Malaysia | Malaysia |
| 15 | PHL | Philippines | Philippines |
| 16 | SGP | Singapore | Singapore |
| 17 | THA | Thailand | Thailand |
| 18 | VNM | Viet Nam | Viet Nam |
| 19 | XSE | Rest of Southeast Asia | Brunei Darussalam, Myanmar, Timor Leste |
| 20 | BGD | Bangladesh | Bangladesh |
| 21 | IND | India | India |
| 22 | NPL | Nepal | Nepal |
| 23 | PAK | Pakistan | Pakistan |
| 24 | LKA | Sri Lanka | Sri Lanka |
| 25 | XSA | Rest of South Asia | Afghanistan, Bhutan, Maldives |
| 26 | CAN | Canada | Canada |
| 27 | USA | United States of America | United States of America |
| 28 | MEX | Mexico | Mexico |
| 29 | XNA | Rest of North America | Bermuda, Greenland, Saint Pierre and Miquelon |
| 30 | ARG | Argentina | Argentina |
| 31 | BOL | Bolivia, Plurinational Republic of | Bolivia, Plurinational Republic of |
| 32 | BRA | Brazil | Brazil |
| 33 | CHL | Chile | Chile |
| 34 | COL | Colombia | Colombia |
| 35 | ECU | Ecuador | Ecuador |
| 36 | PRY | Paraguay | Paraguay |
| 37 | PER | Peru | Peru |
| 38 | URY | Uruguay | Uruguay |
| 39 | VEN | Venezuela | Venezuela |
| 40 | XSM | Rest of South America | Falkland Islands (Malvinas), French Guiana, Guyana, Suriname, South Georgia and the South Sandwich Islands |
| 41 | CRI | Costa Rica | Costa Rica |
| 42 | GTM | Guatemala | Guatemala |
| 43 | HND | Honduras | Honduras |
| 44 | NIC | Nicaragua | Nicaragua |
| 45 | PAN | Panama | Panama |
| 46 | SLV | El Salvador | El Salvador |
| 47 | XCA | Rest of Central America | Belize |
| 48 | XCB | Caribbean | Aruba, Anguilla, Netherlands Antilles, Antigua & Barbuda, Bahamas, Barbados, Cuba, Cayman Islands, Dominica, Dominican Republic, Grenada, Haiti, Jamaica, Saint Kitts and Nevis, Saint Lucia, Montserrat, Puerto Rico, Turks and Caicos Islands, Trinidad and Tobago, Saint Vincent and the Grenadines, Virgin Islands British, Virgin Islands U.S. |
| 49 | AUT | Austria | Austria |
| 50 | BEL | Belgium | Belgium |
| 51 | CYP | Cyprus | Cyprus |
| 52 | CZE | Czech Republic | Czech Republic |
| 53 | DNK | Denmark | Denmark |
| 54 | EST | Estonia | Estonia |
| 55 | FIN | Finland | Finland, Aland Islands |
| 56 | FRA | France | France, Guadeloupe, Martinique, Reunion |
| 57 | DEU | Germany | Germany |
| 58 | GRC | Greece | Greece |
| 59 | HUN | Hungary | Hungary |
| 60 | IRL | Ireland | Ireland |
| 61 | ITA | Italy | Italy |
| 62 | LVA | Latvia | Latvia |
| 63 | LTU | Lithuania | Lithuania |
| 64 | LUX | Luxembourg | Luxembourg |
| 65 | MLT | Malta | Malta |
| 66 | NLD | Netherlands | Netherlands |
| 67 | POL | Poland | Poland |
| 68 | PRT | Portugal | Portugal |
| 69 | SVK | Slovakia | Slovakia |
| 70 | SVN | Slovenia | Slovenia |
| 71 | ESP | Spain | Spain |
| 72 | SWE | Sweden | Sweden |
| 73 | GBR | United Kingdom | United Kingdom |
| 74 | CHE | Switzerland | Switzerland |
| 75 | NOR | Norway | Norway, Svalbard and Jan Mayen |
| 76 | XEF | Rest of EFTA | Iceland, Liechtenstein |
| 77 | ALB | Albania | Albania |
| 78 | BGR | Bulgaria | Bulgaria |
| 79 | BLR | Belarus | Belarus |
| 80 | HRV | Croatia | Croatia |
| 81 | ROU | Romania | Romania |
| 82 | RUS | Russian Federation | Russian Federation |
| 83 | UKR | Ukraine | Ukraine |
| 84 | XEE | Rest of Eastern Europe | Moldova Republic of |
| 85 | XER | Rest of Europe | Andorra, Bosnia and Herzegovina, Faroe Islands, Gibraltar, Monaco, Macedonia the former Yugoslav Republic of, San Marino, Serbia, Guernsey, Isle of Man, Jersey, Montenegro, Holy See (Vatican City State) |
| 86 | KAZ | Kazakhstan | Kazakhstan |
| 87 | KGZ | Kyrgyzstan | Kyrgyzstan |
| 88 | XSU | Rest of Former Soviet Union | Tajikistan, Turkmenistan, Uzbekistan |
| 89 | ARM | Armenia | Armenia |
| 90 | AZE | Azerbaijan | Azerbaijan |
| 91 | GEO | Georgia | Georgia |
| 92 | BHR | Bahrain | Bahrain |
| 93 | IRN | Iran Islamic Republic of | Iran Islamic Republic of |
| 94 | ISR | Israel | Israel |
| 95 | KWT | Kuwait | Kuwait |
| 96 | OMN | Oman | Oman |
| 97 | QAT | Qatar | Qatar |
| 98 | SAU | Saudi Arabia | Saudi Arabia |
| 99 | TUR | Turkey | Turkey |
| 100 | ARE | United Arab Emirates | United Arab Emirates |
| 101 | XWS | Rest of Western Asia | Iraq, Jordan, Lebanon, Palestinian Territory Occupied, Syrian Arab Republic, Yemen |
| 102 | EGY | Egypt | Egypt |
| 103 | MAR | Morocco | Morocco |
| 104 | TUN | Tunisia | Tunisia |
| 105 | XNF | Rest of North Africa | Algeria, Libyan Arab Jamahiriya, Western Sahara |
| 106 | BEN | Benin | Benin |
| 107 | BUF | Burkina Faso | Burkina Faso |
| 108 | CMR | Cameroon | Cameroon |
| 109 | CIV | Cote d'Ivoire | Cote d'Ivoire |
| 110 | GHA | Ghana | Ghana |
| 111 | GUI | Guinea | Guinea |
| 112 | NGA | Nigeria | Nigeria |
| 113 | SEN | Senegal | Senegal |
| 114 | TOG | Togo | Togo |
| 115 | XWF | Rest of Western Africa | Cape Verde, Gambia, Guinea-Bissau, Liberia, Mali, Mauritania, Niger, Saint Helena, Ascension and Tristan Da Cunha, Sierra Leone |
| 116 | XCF | Central Africa | Central African Republic, Congo, Gabon, Equatorial Guinea, Sao Tome and Principe, Chad |
| 117 | XAC | South Central Africa | Angola, Congo the Democratic Republic of the |
| 118 | ETH | Ethiopia | Ethiopia |
| 119 | KEN | Kenya | Kenya |
| 120 | MDG | Madagascar | Madagascar |
| 121 | MWI | Malawi | Malawi |
| 122 | MUS | Mauritius | Mauritius |
| 123 | MOZ | Mozambique | Mozambique |
| 124 | RWA | Rwanda | Rwanda |
| 125 | TZA | Tanzania United Republic of | Tanzania United Republic of |
| 126 | UGA | Uganda | Uganda |
| 127 | ZMB | Zambia | Zambia |
| 128 | ZWE | Zimbabwe | Zimbabwe |
| 129 | XEC | Rest of Eastern Africa | Burundi, Comoros, Djibouti, Eritrea, Mayotte, Sudan, Somalia, Seychelles |
| 130 | BWA | Botswana | Botswana |
| 131 | NAM | Namibia | Namibia |
| 132 | ZAF | South Africa | South Africa |
| 133 | XSC | Customs Union | Lesotho, Swaziland |
| 134 | XTW | Rest of the World | Antarctica, French Southern Territories, Bouvet Island, British Indian Ocean Territory |

| **Table S2.** Definition of sectors. | | |
| --- | --- | --- |
| Number | Sector | Category |
| 1 | Paddy rice | Agriculture |
| 2 | Wheat |  |
| 3 | Cereal grains nec |  |
| 4 | Vegetables, fruit, nuts; |  |
| 5 | Oil seeds |  |
| 6 | Sugarcane, sugar beet |  |
| 7 | Plant-based ﬁbers |  |
| 8 | Crops nec |  |
| 9 | Cattle, sheep, goats, horses |  |
| 10 | Animal products nec |  |
| 11 | Raw milk |  |
| 12 | Wool, silk-worm cocoons |  |
| 13 | Forestry |  |
| 14 | Fishing |  |
| 15 | Coal | Mining |
| 16 | Oil |  |
| 17 | Gas |  |
| 18 | Minerals nec |  |
| 19 | Meat: cattle, sheep, goats, horses | Light Manufacting |
| 20 | Meat products nec |  |
| 21 | Vegetable oils and fats |  |
| 22 | Dairy products |  |
| 23 | Processed rice |  |
| 24 | Sugar |  |
| 25 | Food products nec |  |
| 26 | Beverages and tobacco products |  |
| 27 | Textiles |  |
| 28 | Wearing apparel |  |
| 29 | Leather products |  |
| 30 | Wood products |  |
| 31 | Paper products, publishing |  |
| 32 | Petroleum, coal products | Petroleum, coal products |
| 33 | Chemical, rubber, plastic products | Chemical, rubber, plastic products |
| 34 | Mineral products nec | Mineral products |
| 35 | Ferrous metals |  |
| 36 | Metals nec | Metal products |
| 37 | Metal products |  |
| 38 | Motor vehicles and parts | Equipment Manufacturing |
| 39 | Transport equipment nec |  |
| 40 | Electronic equipment |  |
| 41 | Machinery and equipment nec |  |
| 42 | Manufactures nec |  |
| 43 | Electricity | Power generation |
| 44 | Gas manufacture, distribution |  |
| 45 | Water collection, puriﬁcation, and distribution |  |
| 46 | Construction | Construction |
| 47 | Trade | Non-transport Services |
| 48 | Transport nec | Transport |
| 49 | Sea transport |  |
| 50 | Air transport |  |
| 51 | Communication | Non-transport Services |
| 52 | Financial services nec |  |
| 53 | Insurance |  |
| 54 | Business services nec |  |
| 55 | Recreation and other services |  |
| 56 | Public Administration, Defense, Health, Education |  |
| 57 | Dwellings |  |

**Table S3**. Produced, consumed and traded PM_2.5_ emissions.

| Number | Region | Production based  Emissions (Gg) | Consumption  based Emissions (Gg) | Emission embodied in exports (Gg) | Emission  Embodied in imports (Gg) |
| --- | --- | --- | --- | --- | --- |
| 1 | Australia | 114.5 | 173.4 | 48.6 | 107.5 |
| 2 | New Zealand | 12.4 | 24.5 | 4.2 | 16.4 |
| 3 | Rest of Oceania | 22.9 | 16.8 | 11.2 | 5.1 |
| 4 | The mainland of China | 9371.7 | 6947.7 | 2717.0 | 293.0 |
| 5 | Hong Kong | 0.0 | 80.3 | 0.0 | 80.3 |
| 6 | Japan | 477.0 | 725.2 | 166.9 | 415.1 |
| 7 | Korea | 174.6 | 294.6 | 73.6 | 193.6 |
| 8 | Mongolia | 11.2 | 10.4 | 3.4 | 2.6 |
| 9 | Taiwan | 0.0 | 66.7 | 0.0 | 66.7 |
| 10 | Rest of East Asia | 123.3 | 91.8 | 42.7 | 11.2 |
| 11 | Cambodia | 22.6 | 25.1 | 4.3 | 6.7 |
| 12 | Indonesia | 425.4 | 400.6 | 105.2 | 80.4 |
| 13 | Lao People’s Democratic Republic | 34.0 | 28.8 | 7.5 | 2.2 |
| 14 | Malaysia | 113.3 | 93.4 | 72.2 | 52.4 |
| 15 | Philippines | 116.1 | 118.1 | 28.9 | 30.9 |
| 16 | Singapore | 4.8 | 43.2 | 3.4 | 41.7 |
| 17 | Thailand | 259.3 | 189.3 | 128.0 | 58.0 |
| 18 | Viet Nam | 231.2 | 187.2 | 93.8 | 49.8 |
| 19 | Rest of Southeast Asia | 96.3 | 73.6 | 32.5 | 9.8 |
| 20 | Bangladesh | 164.3 | 179.6 | 10.4 | 25.6 |
| 21 | India | 3060.3 | 2782.3 | 482.8 | 204.8 |
| 22 | Nepal | 29.8 | 32.7 | 2.2 | 5.1 |
| 23 | Pakistan | 251.7 | 256.4 | 32.2 | 36.9 |
| 24 | Sri Lanka | 20.0 | 29.7 | 4.3 | 13.9 |
| 25 | Rest of South Asia | 30.4 | 29.7 | 10.1 | 9.5 |
| 26 | Canada | 147.8 | 219.6 | 76.5 | 148.2 |
| 27 | United States of America | 1217.2 | 2275.4 | 220.2 | 1278.5 |
| 28 | Mexico | 183.1 | 221.3 | 54.6 | 92.8 |
| 29 | Rest of North America | 19.6 | 12.3 | 10.4 | 3.1 |
| 30 | Argentina | 253.1 | 98.3 | 183.0 | 28.2 |
| 31 | Bolivia | 14.0 | 13.7 | 3.2 | 3.0 |
| 32 | Brazil | 678.0 | 567.6 | 211.2 | 100.8 |
| 33 | Chile | 40.0 | 43.4 | 21.8 | 25.2 |
| 34 | Colombia | 53.4 | 64.4 | 13.6 | 24.5 |
| 35 | Ecuador | 21.0 | 27.6 | 4.2 | 10.8 |
| 36 | Paraguay | 27.3 | 18.3 | 13.0 | 4.0 |
| 37 | Peru | 36.3 | 43.6 | 9.4 | 16.8 |
| 38 | Uruguay | 10.0 | 8.5 | 6.3 | 4.8 |
| 39 | Venezuela | 72.0 | 78.1 | 22.2 | 28.2 |
| 40 | Rest of South America | 26.4 | 21.4 | 6.4 | 1.3 |
| 41 | Costa Rica | 6.0 | 7.6 | 3.2 | 4.8 |
| 42 | Guatemala | 11.3 | 14.5 | 3.8 | 7.0 |
| 43 | Honduras | 7.1 | 9.2 | 1.8 | 3.9 |
| 44 | Nicaragua | 3.9 | 5.2 | 1.3 | 2.6 |
| 45 | Panama | 4.5 | 8.4 | 0.7 | 4.7 |
| 46 | EI Salvador | 6.3 | 9.3 | 1.6 | 4.6 |
| 47 | Rest of Central America | 0.4 | 0.6 | 0.2 | 0.4 |
| 48 | Caribbean | 211.7 | 211.8 | 43.2 | 43.4 |
| 49 | Austria | 28.0 | 50.0 | 16.8 | 38.8 |
| 50 | Belgium | 32.6 | 104.6 | 24.1 | 96.2 |
| 51 | Cyprus | 2.8 | 8.8 | 0.7 | 6.6 |
| 52 | Czech Republic | 34.6 | 41.9 | 19.7 | 27.0 |
| 53 | Denmark | 16.6 | 48.5 | 8.5 | 40.4 |
| 54 | Estonia | 11.3 | 13.1 | 4.5 | 6.4 |
| 55 | Finland | 28.4 | 47.3 | 14.3 | 33.2 |
| 56 | France | 141.0 | 318.6 | 53.3 | 230.8 |
| 57 | Germany | 197.0 | 474.9 | 100.6 | 378.5 |
| 58 | Greece | 33.5 | 92.7 | 7.8 | 67.0 |
| 59 | Hungary | 18.7 | 31.5 | 10.2 | 23.0 |
| 60 | Ireland | 10.4 | 33.9 | 5.2 | 28.7 |
| 61 | Italy | 112.9 | 316.2 | 42.5 | 245.7 |
| 62 | Latvia | 6.8 | 12.7 | 3.5 | 9.5 |
| 63 | Lithuania | 15.9 | 20.3 | 7.8 | 12.2 |
| 64 | Luxembourg | 3.2 | 7.4 | 2.5 | 6.7 |
| 65 | Malta | 0.4 | 2.7 | 0.2 | 2.6 |
| 66 | Netherlands | 30.0 | 97.9 | 20.8 | 88.6 |
| 67 | Poland | 149.5 | 157.8 | 56.3 | 64.6 |
| 68 | Portugal | 21.0 | 43.5 | 7.4 | 29.9 |
| 69 | Slovakia | 27.8 | 26.1 | 16.8 | 15.1 |
| 70 | Slovenia | 5.8 | 11.3 | 3.2 | 8.8 |
| 71 | Spain | 124.3 | 276.3 | 40.2 | 192.2 |
| 72 | Sweden | 28.8 | 57.9 | 17.1 | 46.2 |
| 73 | United Kingdom | 105.4 | 377.4 | 37.1 | 309.1 |
| 74 | Switzerland | 12.8 | 52.0 | 6.6 | 45.7 |
| 75 | Norway | 10.2 | 39.2 | 5.9 | 34.9 |
| 76 | Rest of EFTA | 1.4 | 4.7 | 0.7 | 3.9 |
| 77 | Albania | 3.4 | 7.0 | 0.9 | 4.5 |
| 78 | Bulgaria | 36.5 | 32.7 | 17.8 | 14.0 |
| 79 | Belarus | 56.8 | 37.4 | 40.0 | 20.7 |
| 80 | Croatia | 11.5 | 19.3 | 4.3 | 12.1 |
| 81 | Romania | 70.7 | 79.8 | 22.7 | 31.8 |
| 82 | Russian Federation | 907.6 | 791.4 | 339.6 | 223.4 |
| 83 | Ukraine | 357.4 | 166.0 | 239.8 | 48.4 |
| 84 | Rest of Eastern Europe | 4.1 | 7.2 | 2.2 | 5.2 |
| 85 | Rest of Europe | 67.2 | 67.6 | 19.6 | 20.0 |
| 86 | Kazakhstan | 202.5 | 183.0 | 68.9 | 49.5 |
| 87 | Kyrgyzstan | 7.9 | 7.6 | 3.5 | 3.2 |
| 88 | Rest of Former Soviet Union | 67.4 | 53.6 | 29.4 | 15.6 |
| 89 | Armenia | 3.3 | 5.1 | 1.5 | 3.3 |
| 90 | Azerbaijan | 13.1 | 22.1 | 3.2 | 12.2 |
| 91 | Georgia | 4.7 | 8.3 | 2.3 | 5.9 |
| 92 | Bahrain | 1.7 | 5.7 | 0.9 | 4.9 |
| 93 | Iran, Islamic Republic of | 246.7 | 297.9 | 43.0 | 94.2 |
| 94 | Israel | 19.1 | 40.1 | 8.1 | 29.1 |
| 95 | Kuwait | 14.9 | 37.3 | 4.1 | 26.5 |
| 96 | Oman | 8.7 | 20.6 | 2.3 | 14.2 |
| 97 | Qatar | 16.1 | 29.4 | 6.5 | 19.9 |
| 98 | Saudi Arabia | 87.8 | 153.0 | 23.6 | 88.8 |
| 99 | Turkey | 342.8 | 385.6 | 88.6 | 131.3 |
| 100 | United Arab Emirates | 34.7 | 165.1 | 8.4 | 138.8 |
| 101 | Rest of West Asia | 254.5 | 207.7 | 107.5 | 60.6 |
| 102 | Egypt | 302.0 | 256.0 | 85.9 | 40.0 |
| 103 | Morocco | 97.5 | 104.4 | 18.7 | 25.6 |
| 104 | Tunisia | 38.6 | 24.4 | 25.9 | 11.8 |
| 105 | Rest of North Africa | 112.4 | 130.8 | 31.3 | 49.7 |
| 106 | Benin | 7.0 | 8.7 | 5.3 | 7.1 |
| 107 | Burkina Faso | 9.2 | 10.1 | 1.3 | 2.3 |
| 108 | Cameroon | 9.1 | 9.3 | 2.8 | 3.0 |
| 109 | Cote d'lvoire | 4.8 | 8.3 | 1.7 | 5.2 |
| 110 | Ghana | 15.3 | 25.5 | 3.5 | 13.8 |
| 111 | Guinea | 15.2 | 13.7 | 3.4 | 1.9 |
| 112 | Nigeria | 59.5 | 73.1 | 22.6 | 36.1 |
| 113 | Senegal | 15.7 | 15.6 | 5.7 | 5.7 |
| 114 | Togo | 4.4 | 6.4 | 2.9 | 4.9 |
| 115 | Rest of Western Africa | 34.6 | 45.0 | 9.9 | 20.3 |
| 116 | Rest of Central Africa | 12.7 | 18.7 | 4.1 | 10.2 |
| 117 | Rest of South Central Africa | 21.5 | 29.3 | 9.0 | 16.8 |
| 118 | Ethiopia | 25.0 | 32.3 | 1.9 | 9.1 |
| 119 | Kenya | 21.6 | 25.6 | 6.5 | 10.5 |
| 120 | Madagascar | 53.9 | 47.7 | 8.7 | 2.5 |
| 121 | Malawi | 50.6 | 39.8 | 13.0 | 2.2 |
| 122 | Mauritius | 2.2 | 5.1 | 0.9 | 3.7 |
| 123 | Mozambique | 10.0 | 12.5 | 3.9 | 6.4 |
| 124 | Rwanda | 8.6 | 10.1 | 1.3 | 2.8 |
| 125 | Tanzania, United Republic of | 18.3 | 20.2 | 5.5 | 7.4 |
| 126 | Uganda | 103.6 | 88.5 | 19.0 | 3.9 |
| 127 | Zambia | 6.5 | 9.2 | 3.2 | 5.9 |
| 128 | Zimbabwe | 22.5 | 16.0 | 12.3 | 5.8 |
| 129 | Rest of Eastern Africa | 19.4 | 35.7 | 4.1 | 20.4 |
| 130 | Botswana | 4.9 | 11.5 | 2.3 | 8.9 |
| 131 | Namibia | 3.8 | 8.1 | 2.4 | 6.6 |
| 132 | South Africa | 617.4 | 376.8 | 294.2 | 53.7 |
| 133 | Rest of South African Customs Union | 4.9 | 3.7 | 2.2 | 0.9 |
| 134 | Rest of the world | 0.0 | 0.0 | 0.0 | 0.0 |
|  | Total | 24002.4 | 24002.4 | 7236.3 | 7236.3 |

**Table S4.** Budget of average primary fine aerosol concentrations from inter-continental transport of aerosols from each source region to each receptor region, contributions of a region on itself (referred to ‘domestic’) are shown in bold: (a) induced by production of source region [Unit: μg∙m^-3^]; (b) induced by consumption of source region [Unit: μg∙m^-3^]; (c) the ratio concentrations induced by consumption to production.

1. induced by production of source region [Unit: μg∙m^-3^]

| Source Region | Receptor Region | | | |
| --- | --- | --- | --- | --- |
|  | NA | EU | IN | EA |
| NA | **0.349** | 0.007 | 0.002 | 0.002 |
| EU | 0.001 | **0.773** | 0.005 | 0.008 |
| IN | 0.001 | 0.001 | **2.931** | 0.070 |
| EA | 0.012 | 0.004 | 0.019 | **4.263** |

1. induced by consumption of source region [Unit: μg∙m^-3^]

| Source Region | Receptor Region | | | |
| --- | --- | --- | --- | --- |
|  | NA | EU | IN | EA |
| NA | **0.299** | 0.034 | 0.097 | 0.428 |
| EU | 0.017 | **0.631** | 0.121 | 0.383 |
| IN | 0.001 | 0.004 | **2.469** | 0.044 |
| EA | 0.013 | 0.019 | 0.057 | **4.094** |

1. the ratio concentrations induced by consumption to production.

| Source Region | Receptor Region | | | |
| --- | --- | --- | --- | --- |
|  | NA | EU | IN | EA |
| NA | **0.9** | 5 | 40 | 223 |
| EU | 14 | **0.8** | 27 | 50 |
| IN | 1 | 7 | **0.8** | 0.6 |
| EA | 1 | 5 | 3 | **1** |

Figure S1. Distribution of consumption-based PM_2.5_ emissions (F_Cr_) in 2007 by mass of emissions in the region.

Tg/y


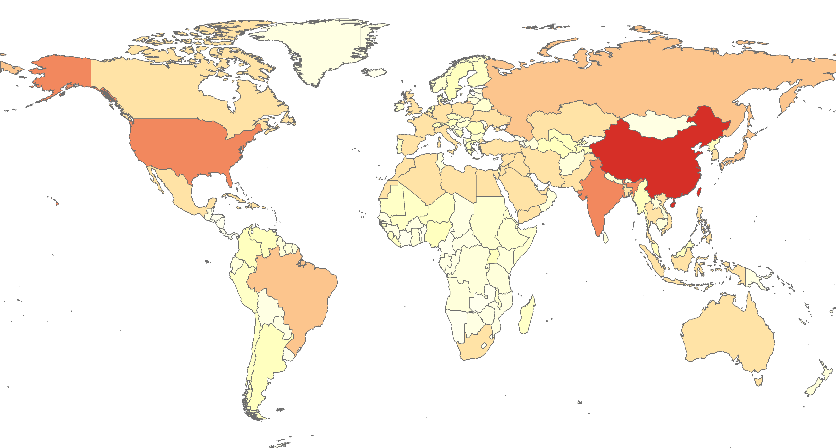





Figure S2. PM_2.5_ emissions embodied in imports and exports of the largest net importing/exporting countries/regions. Intermediate goods (gray) are those used by industries in the importing country to further processing and meet consumer demand for domestic goods.

### Bridging PKU-inventory and GTAP classifications

Bridging the two databases was done by setting up concordance matrices between the 78 emission sources and each of GTAP’s countries’ sectors. Let C be such a concordance matrix, holding C_ij_=1 if PKU-inventory class I corresponds to GTAP sector j, as shown in Table S1.

The original primary fuel type in GTAP database covers coal, oil, coal and oil products, and gas. As the emission factors of various types of fuel varies significant, the coal, oil, coal and oil products were split into seven types of fuel according to the energy statistics from International Energy Agency [1-5]. The coal consumption was divided into: (1) Briquettes, coke; (2) Brown coal, peat; (3) Hard coal, coking coal. The consumption of coal and oil products was divided into: (1) Heavy fuel oil; (2) Gasoline, aviation fuel, kerosene; (3) Liquefied Petroleum Gas. For some developing countries without country-specific energy statistics, average proportion of different types of fuels of NON-OECD countries were used. Then the emissions from power generation were split into 2 sectors (Sector 43-44 in Table S5) according to the sectors’ fuel consumption. For example, the emissions from coke used in power generation were C, the coke consumption in Sector 43 and Sector 44 was E1 and E2, then the emissions from coke comsumption in Sector 43 and Sector 44 was $C*E1/(\mathrm{E}1+\mathrm{E}2)$ and $C*E2/(\mathrm{E}1+\mathrm{E}2)$, respectively. Similarly, emissions from energy combustion in agriculture, industry, residential were split into 14 sectors (Sector 1-14 in Table S1), 30 sectors (Sector 15-42, 45-46 in Table S1), 8 sectors (Sector 47, 51-57 in Table S1) respectively. The emissions from industrial process were attributed to the corresponding sectors of the products.

**Table S5. PKU Emissions Classification to GTAP database aggregated categories**

| Code  name | Name | Power coal | Power oil | Power gas | Power  Coal and oil product | Industry coal | Industry oil | Industry gas | Industry Coal and oil product | Industrial Process | Agriculture | Residential | Transportation |
| --- | --- | --- | --- | --- | --- | --- | --- | --- | --- | --- | --- | --- | --- |
| PKU-source 1 | Anthracite used in power stations | 1 | - | - | - | - | - | - | - | - | - | - | - |
| PKU-source 2 | Coke used in power stations | 1 | - | - | - | - | - | - | - | - | - | - | - |
| PKU-source 3 | Bituminous coal used in power stations | 1 | - | - | - | - | - | - | - | - | - | - | - |
| PKU-source 4 | Lignite used in power stations | 1 | - | - | - | - | - | - | - | - | - | - | - |
| PKU-source 5 | Peat used in power stations | 1 | - | - | - | - | - | - | - | - | - | - | - |
| PKU-source 6 | Gas/Diesel used in power stations | - | - | - | 1 | - | - | - | - | - | - | - | - |
| PKU-source 7 | Residue fuel oil used in power stations | - | 1 | - | - | - | - | - | - | - | - | - | - |
| PKU-source 8 | Solid biomass used in power stations | - | - | - | 1 | - | - | - | - | - | - | - | - |
| PKU-source 9 | Biogas used in power stations | - | - | - | 1 | - | - | - | - | - | - | - | - |
| PKU-source 10 | Municipal waste used in power stations | - | - | - | 1 | - | - | - | - | - | - | - | - |
| PKU-source 11 | Industrial waste used in power stations | - | - | - | 1 | - | - | - | - | - | - | - | - |
| PKU-source 12 | Dry natural gas used in power stations | - | - | 1 | - | - | - | - | - | - | - | - | - |
| PKU-source 13 | Natural gas liquid used in power stations | - | - | - | 1 | - | - | - | - | - | - | - | - |
| PKU-source 14 | Bituminous coal consumed in coke production | - | - | - | - | - | - | - | - | 1 | - | - | - |
| PKU-source 15 | Bituminous coal consumed in brick production | - | - | - | - | - | - | - | - | 1 | - | - | - |
| PKU-source 16 | Anthracite consumed in aluminum production | - | - | - | - | - | - | - | - | 1 | - | - | - |
| PKU-source 17 | Hydraulic Cement Production | - | - | - | - | - | - | - | - | 1 | - | - | - |
| PKU-source 18 | Gas flaring | - | - | - | - | - | - | 1 | - | - | - | - | - |
| PKU-source 19 | Gas/diesel used in agriculture | - | - | - | - | - | - | - | - | - | 1 | - | - |
| PKU-source 20 | Gas/diesel used in industry | - | - | - | - | - | - | 1 | - | - | - | - | - |
| PKU-source 21 | Anthracite used in industry | - | - | - | - | 1 | - |  | - | - | - | - | - |
| PKU-source 22 | Coke used in industry | - | - | - | - | 1 | - | - | - | - | - | - | - |
| PKU-source 23 | Bituminous coal used in industry | - | - | - | - | 1 | - | - | - | - | - | - | - |
| PKU-source 24 | Lignite used in industry | - | - | - | - | 1 | - | - | - | - | - | - | - |
| PKU-source 25 | Peat used in industry | - | - | - | - | 1 | - | - | - | - | - | - | - |
| PKU-source 26 | Residue fuel oil used in industry | - | - | - | - | - | - | - | 1 | - | - | - | - |
| PKU-source 27 | Solid biomass used in industry | - | - | - | - | - | - | - | 1 | - | - | - | - |
| PKU-source 28 | Biogas used in industry | - | - | - | - | - | - | - | 1 | - | - | - | - |
| PKU-source 29 | Municipal waste used in industry | - | - | - | - | - | - | - | 1 | - | - | - | - |
| PKU-source 30 | Industrial waste used in industry | - | - | - | - | - | - | - | 1 | - | - | - | - |
| PKU-source 31 | Crude oil consumed in petroleum refinery | - | - | - | - | - | 1 | - | - | - | - | - | - |
| PKU-source 32 | Dry natural gas used in industry | - | - | - | - | - | - | 1 | - | - | - | - | - |
| PKU-source 33 | Natural gas liquid used in industry | - | - | - | - | - | - | - | 1 | - | - | - | - |
| PKU-source 34 | Small-scaled solid waste burning | - | - | - | - | - | - | - | - | - | - | 1 | - |
| PKU-source 35 | Anthracite used in residential or commercial sector | - | - | - | - | - | - | - | - | - | - | 1 | - |
| PKU-source 36 | Coke used in power in residential or commercial sector | - | - | - | - | - | - | - | - | - | - | 1 | - |
| PKU-source 37 | Bituminous coal used in residential or commercial sector | - | - | - | - | - | - | - | - | - | - | 1 | - |
| PKU-source 38 | Lignite used in residential or commercial sector | - | - | - | - | - | - | - | - | - | - | 1 | - |
| PKU-source 39 | Peat used in residential or commercial sector | - | - | - | - | - | - | - | - | - | - | 1 | - |
| PKU-source 40 | Liquid petroleum gas used in residential or commercial sector | - | - | - | - | - | - | - | - | - | - | 1 | - |
| PKU-source 41 | Dry natural gas used in residential or commercial sector | - | - | - | - | - | - | - | - | - | - | 1 | - |
| PKU-source 42 | Natural gas liquid used in residential or commercial sector | - | - | - | - | - | - | - | - | - | - | 1 | - |
| PKU-source 43 | Kerosene used in residential or commercial sector | - | - | - | - | - | - | - | - | - | - | 1 | - |
| PKU-source 44 | Biogas used in residential or commercial sector | - | - | - | - | - | - | - | - | - | - | 1 | - |
| PKU-source 45 | Firewood used in residential or commercial sector | - | - | - | - | - | - | - | - | - | - | 1 | - |
| PKU-source 46 | Straw used in residential or commercial sector | - | - | - | - | - | - | - | - | - | - | 1 | - |
| PKU-source 47 | Dung cake used in residential or commercial sector | - | - | - | - | - | - | - | - | - | - | 1 | - |
| PKU-source 48 | Motor vehicle gasoline | - | - | - | - | - | - | - | - | - | - | - | 1 |
| PKU-source 49 | Aviation gasoline | - | - | - | - | - | - | - | - | - | - | - | 1 |
| PKU-source 50 | Biodiesel used by vehicles | - | - | - | - | - | - | - | - | - | - | - | 1 |
| PKU-source 51 | Jet kerosene | - | - | - | - | - | - | - | - | - | - | - | 1 |
| PKU-source 52 | Motor vehicle gas/diesel | - | - | - | - | - | - | - | - | - | - | - | 1 |
| PKU-source 53 | Open burning of agriculture waste | - | - | - | - | - | - | - | - | - | 1 | - | - |
| PKU-source 54 | Biomass burned in forest fires | - | - | - | - | - | - | - | - | - | - | - | - |
| PKU-source 55 | Biomass burned in deforestation fires | - | - | - | - | - | - | - | - | - | - | - | - |
| PKU-source 56 | Biomass burned in peat fires | - | - | - | - | - | - | - | - | - | - | - | - |
| PKU-source 57 | Biomass burned in woodland fires | - | - | - | - | - | - | - | - | - | - | - | - |
| PKU-source 58 | Biomass burned in savanna fires | - | - | - | - | - | - | - | - | - | - | - | - |
| PKU-source 59 | Oil used by ocean tanker | - | - | - | - | - | - | - | - | - | - | - | 1 |
| PKU-source 60 | Oil used by ocean container ships | - | - | - | - | - | - | - | - | - | - | - | 1 |
| PKU-source 61 | Oil used by ocean bulk and combined carriers | - | - | - | - | - | - | - | - | - | - | - | 1 |
| PKU-source 62 | Oil used by general cargo vessels | - | - | - | - | - | - | - | - | - | - | - | 1 |
| PKU-source 63 | Oil used by noncargo vessels | - | - | - | - | - | - | - | - | - | - | - | 1 |
| PKU-source 64 | Oil used by auxiliary engines | - | - | - | - | - | - | - | - | - | - | - | 1 |
| PKU-source 65 | Oil used by military vessels | - | - | - | - | - | - | - | - | - | - | - | 1 |
| PKU-source 66 | pig iron | - | - | - | - | - | - | - | - | 1 | - | - | - |
| PKU-source 67 | raw steel | - | - | - | - | - | - | - | - | 1 | - | - | - |
| PKU-source 68 | raw steel | - | - | - | - | - | - | - | - | 1 | - | - | - |
| PKU-source 69 | raw steel | - | - | - | - | - | - | - | - | 1 | - | - | - |
| PKU-source 70 | hot rolled steel | - | - | - | - | - | - | - | - | 1 | - | - | - |
| PKU-source 71 | Catalytic Cracking Capacity | - | - | - | - | - | - | - | - | 1 | - | - | - |
| PKU-source 72 | waste used | - | - | - | - | - | - | - | - | 1 | - | - | - |
| PKU-source 73 | Glass | - | - | - | - | - | - | - | - | 1 | - | - | - |
| PKU-source 74 | Fertilizer | - | - | - | - | - | - | - | - | 1 | - | - | - |
| PKU-source 75 | Ferroalloys | - | - | - | - | - | - | - | - | 1 | - | - | - |
| PKU-source 76 | Lead | - | - | - | - | - | - | - | - | 1 | - | - | - |
| PKU-source 77 | Magnesium | - | - | - | - | - | - | - | - | 1 | - | - | - |
| PKU-source 78 | Zinc | - | - | - | - | - | - | - | - | 1 | - | - | - |

**Reference**

(1). IEA (2010) Energy Statistics of Non‐OECD Countries 2010: (Complete Edition ‐ ISBN 9789264084117). *Sourceoecd Energy* volume 2010(16):i-770(770).

(2). IEA (2012) Energy Statistics of OECD Countries 2012: Complete Edition - ISBN 9789264173736. *Sourceoecd Energy* volume 2012(8):i-426(426).

(3) IEA (2007) Energy Statistics of Non&#8208;OECD Countries: 2004/2005: 2007 Edition (Complete Edition &#8208; ISBN 9264027688). *Source OECD Energy* 2007(15):i-786.

(4) IEA 2010) Energy Statistics of OECD Countries 2010: (Complete Edition ‐ ISBN 9789264084087). *Sourceoecd Energy* volume 2010(16):i-398.

(5) IEA (2010) Energy Statistics of OECD Countries 2010: (Edition complète &#8208; ISBN 9789264084094 &#8208; Fr. à paraître). *SourceOCDE Energie* 2010(26):i-398.
